# How Inclusive, User-Centered Design Research Can Improve Psychological Therapies for Psychosis: Development of SlowMo

**DOI:** 10.2196/11222

**Published:** 2018-12-05

**Authors:** Amy Hardy, Anna Wojdecka, Jonathan West, Ed Matthews, Christopher Golby, Thomas Ward, Natalie D Lopez, Daniel Freeman, Helen Waller, Elizabeth Kuipers, Paul Bebbington, David Fowler, Richard Emsley, Graham Dunn, Philippa Garety

**Affiliations:** 1 Department of Psychology Institute of Psychiatry, Psychology & Neuroscience King's College London London United Kingdom; 2 South London & Maudsley NHS Foundation Trust London United Kingdom; 3 Helen Hamlyn Centre for Design Royal College of Art London United Kingdom; 4 Evolyst Limited Warwick United Kingdom; 5 Department of Psychology Royal Holloway Egham United Kingdom; 6 Department of Psychiatry University of Oxford Oxford United Kingdom; 7 Division of Psychiatry University College London London United Kingdom; 8 Department of Psychology University of Sussex Sussex United Kingdom; 9 Department of Biostatistics and Health Informatics Institute of Psychiatry, Psychology & Neuroscience King's College London London United Kingdom; 10 School of Health Sciences University of Manchester Manchester United Kingdom

**Keywords:** inclusive design, user-centered design, participatory design, design thinking, mHealth, eHealth, digital therapy, schizophrenia, paranoia, psychosis

## Abstract

**Background:**

Real-world implementation of psychological interventions for psychosis is poor. Barriers include therapy being insufficiently usable and useful for a diverse range of people. User-centered, inclusive design approaches could improve the usability of therapy, which may increase uptake, adherence, and effectiveness.

**Objective:**

This study aimed to optimize the usability of an existing psychological intervention, Thinking Well, which targets reasoning processes in paranoia using a basic digital interface.

**Methods:**

We conducted inclusive, user-centered design research characterized by purposive sampling of *extreme* users from the margins of groups, ethnographic investigation of the problem context, and iterative prototyping of solutions. The UK Design Council’s double diamond method was used. This consisted of 4 phases: *discover*, including a case series of Thinking Well, stakeholder interviews, desk research, user profiling, system mapping, and a mood board; *define*, consisting of workshops to synthesize findings and generate the design brief; *develop*, involving concept workshops and prototype testing; and *deliver*, in which the final minimal viable product was storyboarded and iteratively coded.

**Results:**

Consistent with our previous work, the Thinking Well case series showed medium to large effects on paranoia and well-being and small effects on reasoning. These were maintained at follow-up despite some participants reporting difficulties with the therapy interface. Insights from the *discover* phase confirmed that usability was challenged by information complexity and poor accessibility. Participants were generally positive about the potential of technology to be enjoyable, help manage paranoia, and provide tailored interpersonal support from therapists and peers, although they reported privacy and security concerns. The *define* phase highlighted that the therapy redesign should support monitoring, simplify information processing, enhance enjoyment and trust, promote personalization and normalization, and offer flexible interpersonal support. During the *develop* phase over 60 concepts were created, with 2 key concepts of thoughts visualized as bubbles and therapy as a journey selected for storyboarding. The output of the *deliver* phase was a minimal viable product of an innovative digital therapy, SlowMo. SlowMo works by helping people to notice their worries and fast thinking habits, and encourages them to *slow down for a moment* to find ways of feeling safer. A Web app supports the delivery of 8 face-to-face sessions, which are synchronized to a native mobile app.

**Conclusions:**

SlowMo makes use of personalization, ambient information, and visual metaphors to tailor the appeal, engagement, and memorability of therapy to a diversity of needs. Feasibility testing has been promising, and the efficacy of SlowMo therapy is now being tested in a multicentered randomized controlled trial. The study demonstrates that developments in psychological theory and techniques can be enhanced by improving the usability of the therapy interface to optimize its impact in daily life.

## Introduction

### Background to SlowMo Therapy

The development of psychological interventions for psychosis has accelerated in the last 2 decades, particularly with the second- and third-wave cognitive behavioral therapies (CBT) [1]. Although these show promise in reducing distress and improving people’s quality of life, significant barriers to real-world effectiveness remain [2,3]. Effect sizes are in the small to medium range, and psychological interventions are only accessed by 15 to 30% of eligible service users [4-8]. Some people are not motivated to try therapy and those that do may struggle to understand it and to apply new insights to everyday situations [9-11]. Efforts to improve effectiveness have focused so far on identifying causal mechanisms linked to specific outcomes and developing therapy techniques that target these mechanisms [12]. For example, interventions for sleep, worry, self-esteem, and reasoning styles have demonstrated larger effect sizes on paranoia compared with generic CBT for psychosis [13-15]. However, there is continuing concern about barriers to therapy access, uptake, and adherence [5,12,16], and strategies for improving implementation are urgently needed.

We propose that enhancing the usability (or ease-of-use) of therapy will address implementation barriers and thereby improve effectiveness. To the best of our knowledge, this study is the first to focus on optimizing the usability of an existing therapy (Thinking Well) by conducting inclusive, user-centered design (UCD) research. Thinking Well is a brief protocol-based therapy that targets jumping-to-conclusions and belief inflexibility, the reasoning styles that contribute to paranoia [17]. We have already shown that this therapy improved reasoning and reduced paranoia in a case series, a randomized experimental study, and 2 feasibility randomized controlled trials [10,18-20]. However, its effects declined following the end of therapy, and some people reported that the intervention was insufficiently personalized, enjoyable, or applicable to daily life. Moreover, people with working memory problems and negative symptoms tended to benefit less from the therapy [10]. This may, in part, be because of the use of thought records, a widely used tool for the identification and evaluation of distressing cognitions in CBT. Thought records can be cognitively demanding to complete, and their pen-and-paper verbal format limits their usefulness in supporting real-world behavior change [21,22]. Before proceeding to a multicenter randomized controlled trial, we therefore sought to optimize the usability of the intervention to address these concerns. The output of this study, SlowMo, is an innovative blended digital therapy for people who fear harm from others. A Web app supports the delivery of 8 face-to-face sessions, which are synchronized to a native mobile app for use in daily life. SlowMo works by helping people to notice their worries and fast thinking habits, and encourages them to *slow down for a moment* to find ways of feeling safer. SlowMo is currently being tested in a multicenter randomized controlled trial [23].

### Designing Digital Interventions for Psychosis

SlowMo reflects the rapid growth of digital technology in mental health care, given its potential to improve access, outcomes, and costs [24-26]. In psychosis, findings indicate promising rates of acceptability, usability, and safety for interventions delivered via the Web, short message service (SMS) text messaging, mobile phone apps, and virtual reality. However, research is in its infancy and further development and testing are required [27-31]. In addition, gender, age, ethnicity, severity of difficulties, digital literacy, and social support may moderate adherence. This suggests interventions need to be tailored to the needs of a range of potential users [32-36]. Indeed, concerns about uptake and adherence are common in digital health interventions, given the marked overrepresentation among users of highly educated women. This highlights the need to ensure technology interfaces are more compelling and appealing across all groups in society [37].

*Design thinking* is a process whereby challenges to therapy access, uptake, and adherence can be addressed. It involves developing a rich understanding of the problem area and its context to identify valued outcomes. From this, themes are derived to develop possible new ways of *framing* the problem by highlighting its paradoxes, and solutions are then generated to resolve them [38,39]. For example, paradoxes that design thinking may help to resolve include a person’s desire to be healthier while continuing to engage in unhealthy behaviors or government attempts to promote a sense of safety through authoritarian controls that actually exacerbate public perceptions that society is dangerous. However, design thinking alone is insufficient to lead to meaningful change, as professional designers often operate outside problem contexts, and this may limit their ability to understand the problem and develop effective solutions.

UCD methods address this limitation as they privilege the empathic understanding of end users and their contexts, thereby ensuring solutions are relevant to the diverse needs of people involved [39-41]. *Participatory design*, or codesign, is a UCD technique that emphasizes direct user involvement and has its roots in activism and shared decision making. It is increasingly used in digital mental health research, based on ethnographic and qualitative methods [42-44]. To date, participatory design methods used in the development of digital therapies for psychosis have included investigation of stakeholder attitudes through observation, surveys, interviews and focus groups, workshops to develop and test prototype ideas, and laboratory-based *think aloud* usability tests [45-53]. However, these studies have not tended to incorporate design thinking methodology, which can constrain innovation, so that new designs are variations of the status quo. In addition, a risk inherent in participatory design is that the most willing, able, and vocal users are more likely to be involved so that the needs of marginalized people are neglected.

### Research Objectives

Our multidisciplinary collaboration of people with lived experience, clinicians, researchers, industrial designers, and software developers aimed to integrate the best practice principles of design thinking and participatory design. This involved using the Design Council’s [54] double diamond method and adopting an inclusive UCD approach. The double diamond consists of ethnographic investigation of the problem context (the *discover* phase) and using insights from this phase to reframe the problem and generate a design brief (the *define* phase). From this, solutions are generated and iteratively tested with users (the *develop* phase), with feedback determining the optimal design for development (the *deliver* phase). Our strategy for involving people in the design process, inclusive UCD, is different from conventional participatory design. It involves purposive sampling of people at the margins of a normal distribution (extreme users) to ensure the design solution is suitable for the widest range of people. This purposive sampling of extreme users can help to ensure the needs of marginalized groups are considered [55]. On the basis of previous findings, we assumed demographics, cognitive abilities, use of technology, and attitudes to therapy were of particular relevance to the therapy design. We therefore aimed to ensure our sample of people with lived experience of psychosis reflected the extremes of these dimensions.

In summary, we anticipated that the inclusive, UCD research methods employed would support the development of an improved version of the Thinking Well intervention tailored to meet a diversity of needs. Our intention was that the design thinking approach would result in a redesign of the therapy that was more accessible, appealing, memorable, and easy to use, both within sessions and in daily life.

## Methods

### Study Design

We conducted our design research alongside a case series of the previous version of the *Thinking Well* therapy. This was done to support the discovery phase of the double diamond. The case series will first be described, followed by an overview of the double diamond method. The design research was conducted from October 2014 to May 2017.

### Thinking Well Case Series

#### Participants

Fourteen participants were recruited from community mental health teams in a National Health Service (NHS) Trust between March 2014 and May 2015 (see Table 1 in the Results section). Inclusion criteria were a diagnosis of nonaffective psychosis, aged 18 to 65 years at study entry, with relatively stable symptoms and no major crisis in the 3 months before participation, a sufficient level of English to complete measures and participate in the intervention, and a score of 33 or above on the Green Paranoid Thoughts Scale (GPTS) [56]. Exclusion criteria were lack of capacity to provide informed consent, primary diagnosis of substance dependency, and a primary diagnosis of organic syndrome or learning disability.

#### Design

A case series design was used. Assessments were conducted at baseline, post therapy (8 weeks), and at follow-up (12 weeks).

#### Intervention

The case series used the fourth version of Thinking Well, which built on earlier iterations and aimed to incorporate the participant feedback from our previous trial (see Waller et al’s study [20] for a description of the preceding version of the therapy). This new version was developed before starting the inclusive UCD research. The changes included presentation of therapy session materials in PowerPoint, on a laptop, to allow for more multimedia, interactive content; Web pages hosted on a NHS website to support the use of therapy strategies outside of sessions; and use of everyday accessible terminology for key psychological concepts. For example, the terms fast and slow thinking were introduced as a heuristic for capturing the ideas of jumping to conclusions and belief inflexibility, and analytical and reflective thinking, together with the focus on *slowing down for a moment* as a means of managing worries [57]. Other changes, based on feedback from therapists in the previous trial, included extending the therapy content from 4 to 6 meetings and adding sessions on the impact of past experiences and confirmatory bias in paranoia. Although this version of Thinking Well was more digitized than previous versions, the software was not fully interactive. Pen and paper materials were still used during therapy sessions and offered for out-of-session use if people were unwilling or unable to use the Web pages. Screenshots providing an example of the therapy materials, taken from session 1, are shown in Multimedia Appendix 1 (including PowerPoint slides used in the session with images of the paper thought record and practice card and the out-of-session Web pages). Therapy was delivered by clinical psychologists with at least 5 years of experience in delivering cognitive behavioral therapy for psychosis (CBTp) or therapists who had completed a postgraduate CBTp diploma and had a minimum of 1 year of postqualification experience.

#### Measures

##### Positive and Negative Symptoms

The Scale for the Assessment of Positive Symptoms (SAPS) [58] is a 34-item semistructured interview used to assess the severity of hallucinations, delusions, bizarre behavior, and positive formal thought disorder. Each item is rated over the past month from 0 (absent) to 5 (severe) with global ratings for each section. Negative symptoms over the past week were assessed using the Brief Negative Symptom Scale (BNSS), a 13-item semistructured interview measuring blunted affect, alogia, asociality, anhedonia, and avolition, on a 7-point scale from 0 (absent) to 6 (severe) [59]. The SAPS and BNSS were only completed at baseline to assess the clinical characteristics of the sample.

##### State Paranoia

The GPTS [56] is a 32-item measure of state paranoia with sections on ideas of reference and persecution. Each item is rated over the past month from 1 (not at all) to 5 (totally) and a total score is derived.

##### Paranoia Distress and Preoccupation

Participants were asked to rate their current distress and preoccupation regarding their main paranoia belief using a 100-point Visual Analog Scale (VAS) ranging from 0 (not at all) to 100 (totally).

##### Paranoia Conviction

Using a VAS, participants were asked to provide a rating between 0 (believe not at all) and 100 (believe absolutely) of their current conviction in their main paranoia belief.

##### Belief Flexibility

Two items were employed to assess belief flexibility. Possibility of being mistaken was assessed using an item from the Maudsley Assessment of Delusions Scale [60], with participants providing a rating from 0 to 100 to indicate if it was at all possible that they may be mistaken in their belief. The Explanation of Experiences assessment [61] was then used to explore whether participants had any alternative explanations for the experiences contributing to their main paranoia belief.

##### Well-Being

The Warwick-Edinburgh Mental Well-Being Scale [62] was used to measure participants’ sense of well-being. This consists of 14 items, rated from 1 (none of the time) to 5 (all of the time), measuring the degree of positive emotions, fulfilling personal relationships, and sense of agency experienced by participants. A total score is derived, with higher scores indicating more well-being.

##### Therapy Feedback

A semistructured interview schedule was used after each therapy session and at the end of therapy to elicit feedback regarding acceptability, usefulness, and usability.

#### Analysis

Feedback interviews are summarized descriptively. As this case series was primarily conducted to support the design research and not powered to detect significant effects, the focus of the results is not on significance testing. However, to support comparison with our previous work, we report Cohen *d* standardized effect sizes for continuous outcomes, calculated as the difference in the mean between 2 time points divided by the SD of the change.

### Inclusive, User-Centered Design Research

**Figure 1:**
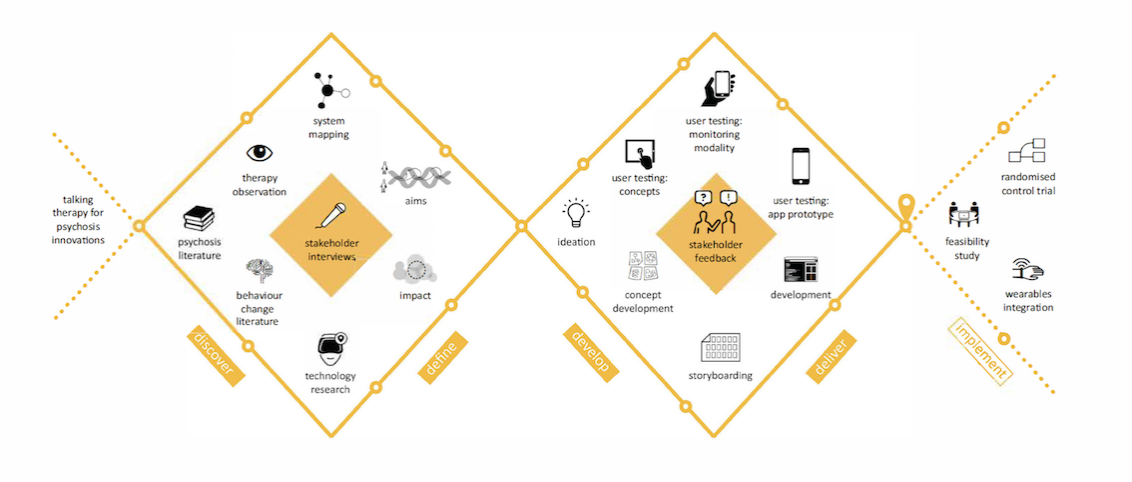
The design research methods used at each phase of the double diamond to develop SlowMo therapy.

An overview of the design research methods used at each phase of the double diamond is shown in Figure 1 and will be further described below.

#### Discover

The aim of this phase was to develop a shared understanding of psychological therapy, behavior change, psychosis, and technology use from the perspective of service users, carers, therapists, and clinicians. This phase is *divergent* in its approach as it explores the subject matter from a variety of viewpoints. It started with desk research covering empirical studies, self-help books, therapy manuals, lived experience narratives, computer games, and gamification. The lead designer (AW) did live and taped observations of the Thinking Well case series and, for comparison, taped observations of 2 cases of a therapy targeting anxiety processes in paranoia. In total, 6 service users were interviewed about topics, including their daily habits, therapy experiences, attitudes toward therapy, and technology preferences. Therapists were shadowed in their service contexts to gain insight into their roles and service user journeys through the system. Following these tasks, user profiles of prototypical service users and therapists were created, together with mapping of the contexts in which therapy is delivered. Methods for illustrating and visualizing thoughts and emotions were also explored to identify the most intuitive ways of communicating them. This included research into areas such as art, visual communication, symbolism, music, movement, and dyslexia.

#### Define

The define phase is *convergent* in its approach, aiming to refine and reframe the breadth of insights emerging from the *discover* phase. This consisted of workshops to synthesize the research insights into themes and identify the most salient areas for improving mental health care in psychosis. A matrix of service users’ and clinicians’ needs in relation to the therapy was developed, with each need rated according to potential impact and ease of implementation. On the basis of these insights, a design brief was developed, specifying the desired area of impact and aims for the redesign.

#### Develop

The develop phase resumed a divergent process, which focused on creating a wide range of ideas for addressing the design brief. At the beginning of the develop phase, concepts to address the design brief were generated, developed, evaluated, and refined by the project designers, technologists, and psychologists. Prototypes of the selected concepts were then made and validated with service users. Prototypes for different modalities of monitoring worries were also explored.

#### Deliver

The *convergent* deliver phase consisted of refining the breadth of concepts generated in the *develop* phase. The selected concepts for therapy redesign were finalized and storyboards were developed. The new version of the design was iteratively produced through rapid prototyping in software code, with user testing of a low fidelity version of the therapy redesign. This resulted in the final minimal viable product.

## Results

### Thinking Well Case Series

Forty-five service users were referred: 12 were unsuitable before screening, 4 declined to meet, 5 did not meet the cut-off score for paranoia, and 24 were suitable. Four service users disengaged between screening and consent, and 20 service users consented to participate. Of those consented, 6 disengaged during the baseline assessment. Fourteen participants were included in the case series, 2 dropped out, and 12 completed the intervention. One of the participants who dropped out experienced a relapse in mental state that was assessed as unrelated to participation in the study and the other disengaged from therapy. No other adverse events were reported. All participants who completed the intervention did the posttherapy assessment and 10 completed the 12-week follow-up assessment. One participant was not contactable as they had moved out of the area and the other was not able to attend because of new personal commitments.

The case series sample demographics are presented in Table 1 and the outcome data and summary statistics in Table 2. Inspection of the descriptive statistics and effect sizes indicates there were improvements in all measures post therapy and at follow-up, relative to baseline. These were in the medium to large range for paranoia and well-being, with small effects on reasoning variables. The results were maintained at follow-up, in contrast to our previous findings [23] where effects reduced at follow-up on all key outcomes. This suggests the extension of the therapy from 4 to 6 sessions was useful, together with an increased focus on multimedia content and normalizing accessible language. Table 3 shows themes arising from the therapy feedback, including experience of the therapy, strategies for feeling safer, and suggestions for improvement, with illustrative quotes. Participants indicated the therapy was helpful in supporting the learning of slow-thinking strategies, and they valued the digital presentation of materials in sessions. They also wanted less verbal information and more interactive and accessible content.

All participants were offered the opportunity to register to the website, which hosted the therapy Web pages. Of the 12 participants in the case series, all expressed an initial interest and 4 completed registration. Of these, 3 never accessed the Web pages and 1 person logged on once, with support from their therapist. Participants were asked about their reasons for not accessing the Web pages at the posttherapy assessment (see Table 4). Responses indicated that the website was too difficult to access because of it only being available on a computer, involving complex log-in instructions, and having an unappealing user interface. This suggested that although people were positive about the use of technology, the basic Web pages were not helpful in improving the therapy experience.

**Table 1:** Thinking Well case series sample demographics (N=12).

Variable		Statistics	Range
Age in years, mean (SD)		43.83 (11.40)	N/A^a^
**Sex, n (%)**			
	Male	5 (42)	N/A
	Female	7 (58)	N/A
**Ethnicity, n (%)**			
	White British	7 (58)	N/A
	Black British	2 (17)	N/A
	Black African	1 (8)	N/A
	Afro-Caribbean	1 (8)	N/A
	Black Caribbean and white	1 (8)	N/A
**Marital status, n (%)**			
	Single	9 (75)	N/A
	Married	3 (25)	N/A
**Employment status, n (%)**			
	Unemployed	8 (68)	N/A
	Carer or housewife	1 (8)	N/A
	Employed	1 (8)	N/A
	Volunteer	1 (8)	N/A
	Student	1 (8)	N/A
**SAPS^b^ positive symptoms, mean (SD)**			
	Hallucinations	2.23 (2.20)	0-5
	Delusions	4.00 (0.58)	3-5
	Bizarre behavior	0.08 (0.28)	0-1
	Formal thought disorder	1.00 (1.16)	0-3
**BNSS^c^ negative symptoms, mean (SD)**			
	Anhedonia	1.18 (1.20)	0-4
	Lack of normal distress	0.31 (0.75)	0-2
	Asociality	1.42 (1.66)	0-6
	Avolition	1.23 (1.28)	0-4
	Blunted affect	1.21 (1.23)	0-5
	Alogia	0.65 (1.11)	0-4

^a^N/A: not applicable.

^b^SAPS: Scale for the Assessment of Positive Symptoms.

^c^BNSS: Brief Negative Symptom Scale.

**Table 2:** Case series paranoia, well-being, and thinking habit outcomes.

Variable	Baseline (n=12)	Post therapy (8 weeks; n=12)		Follow-up (12 weeks; n=10)	
		Statistics	Cohen *d*	Statistics	Cohen *d*
GPTS^a^, mean (SD)	105.50 (17.40)	91.33 (28.49)	0.59	89.90 (37.19)	0.44
VAS^b^ distress, mean (SD)	79.58 (16.16)	61.67 (34.00)	0.61	58.80 (37.30)	0.75
VAS preoccupation, mean (SD)	70.58 (25.46)	62.92 (30.56)	0.50	55.00 (31.97)	0.75
WEMWBS^c^, mean (SD)	39.13 (2.80)	42.55 (7.84)	0.71	43.22 (9.38)	0.40
VAS conviction, mean (SD)	75.42 (29.65)	56.83 (32.91)	0.67	55.00 (37.11)	0.63
VAS possibility of being mistaken^d^, mean (SD)	36.36 (37.69)	41.75 (35.78)	0.12	46.50 (34.32)	0.20
n with ≥1 alternative explanations, n (%)	4 (33)	6 (50)	N/A^e^	8 (80)	N/A

^a^GPTS: Green Paranoid Thoughts Scale.

^b^VAS: Visual Analog Scale.

^c^WEMWBS: Warwick-Edinburgh Mental Well-Being Scale; baseline: n=8, post: n=11, follow-up: n=9.

^d^Baseline: n=11.

^e^N/A: not applicable.

**Table 3:** Case series therapy feedback.

Theme	Comments
Experience of therapy interface	“More helpful than talking therapy because it had the computer programme. I felt comfortable rather than worried I wouldn’t know what to say.” “Videos, liked the visual representation of how events can change mood and thinking.” “Comfortable. I’m not too good at talking but with someone who knows what they’re talking about it helps bring it out.” “Don’t like the writing—I prefer the therapist to write.” “I found it quite hard because I had to think more.”
Strategies for feeling safer	“Using the coping cards, photographing them so I have them on my phone. Trying to practise to keep it in mind.” “Looking for evidence, trying to think outside the box and looking for alternatives.” “Slowing down and thinking that it could be something else.” “Dwelling less, doing more with friends and family, slowing down, and looking for more information.” “The suspicions come up, but they don’t escalate cause I’ve got tools I can reach for.”
Suggestions for improvement	“More videos—they are a good visual aid and more relatable.” “Getting people together to say what they’ve learnt, even just at the end.” “Oyster card wallet that contains the cards to help people remember the coping strategies.” “More interactive things and more interactive scenarios to help practise other explanations.” “Examples of other people’s past experiences and how they affect them.”

**Table 4:** Case series Web pages feedback.

Theme	Comments
Hardware accessibility	“They were too difficult to access, the website was only available on a computer and I don’t have one.” “It was too much effort to go to the drop-in sessions that the trust hosted to use the website.”
Software accessibility	“It meant finding the handouts, getting to a computer, and writing in the address to access the website, as well as a number of instructions just with the welcome pack, it’s asking a lot of effort.” “The password got sent separately by post, I lost it.” “It was difficult to remember how to use.”
User interface	“Interface was not user friendly or self-explanatory. Finding things on the page was difficult even once I’d managed to login.”

### Design Research

The key insights and outputs from each phase of the double diamond will be described below. There were 18 participants in the design research sample. The sample included all the participants who completed the case series, the participants who disengaged from the case series, and 5 participants who were purposively recruited to improve the extent to which the sample represented the extremes of our target sampling characteristics (ie, demographics, digital literacy, cognitive abilities, and relationship to therapy). The sample included 9 (9/18, 50%) men and 9 (9/18, 50%) women (age range 23-62 years). Seven (7/18, 39%) participants were white British, 3 were black Caribbean (3/18, 17%), 2 were black African (2/18, 11%), 2 were black British (2/18, 11%), 2 were white British and black Caribbean (2/18, 11%), 1 was white British and black African (1/18, 6%), and 1 was white British and black Caribbean (1/18, 6%). On the basis of their self-report and presentation during the design research tasks, 2 (2/18, 11%) participants appeared to have above-average cognitive abilities, 7 (7/18, 39%) participants had average cognitive abilities, 5 (5/18, 28%) participants had mild difficulties with attention, reasoning, and memory (often because of psychotic experiences), and 4 (4/18, 22%) participants had moderate to severe difficulties in these areas. In relation to digital literacy, 2 (2/18, 11%) participants had minimal experience of using technology, of whom 1 was interested in developing their skills and 1 was not. Seven (7/18, 39%) participants used a basic mobile phone, of whom 3 were not confident in using. Nine (9/18, 50%) participants had experience with smartphones and laptops, including 7 frequent and competent users and 2 who were not confident in using and wished to improve their skills. With regard to attitudes to therapy, 11 (11/18, 61%) participants viewed therapy as both supportive and useful. In addition, 4 of these reported no difficulty in applying insights to daily life and 7 reported struggling to generalize strategies outside of therapy because of the intensity of their distress, memory problems, motivation, social stressors, and physical health problems. The remaining 7 (7/18, 39%) participants were ambivalent about therapy usefulness either because they were unsure of its relevance to their problems or struggled with its reliance on verbal material and paper tools. Involvement in the design research tasks varied across participants, 6 participants were interviewed, 15 had either live or taped observations of their therapy sessions, and 4 participants were involved in prototype testing.

#### Discover

Multimedia Appendix 2 contains the processes and outputs during the discover phase, including process map of therapy sessions, mapping of the broader multidisciplinary service context, service user journeys, user profiles, a mood board reflecting the communication of thoughts and emotions, and a table summarizing the 5 salient themes arising from this phase, illustrated by comments from the participant interviews. These 5 themes were validated against the insights arising from the therapy observations, service shadowing, and context mapping.

The first theme concerned challenges to the usability of therapy. Service users and therapists struggled to manage information processing and communication demands, given the amount and complexity of the therapy materials. This limited the potential impact of therapy on people’s lives. As a result, adaptations were made to make the materials more concise and accessible, such as personalizing the content and using mobile phones to record therapy strategies. The second theme related to technology use. Concerns about digital literacy and privacy were frequent, although these often occurred alongside a desire to integrate technology into therapy and improve digital skills. A wish to progress and to document achievements using technology was also highlighted. Enjoyment was the focus of the third theme, with a consensus that interactive, gamified tasks and visual materials were the most enjoyable aspects of therapy. The next theme related to the therapy relationship. Feedback in this area reflected some people valuing the support from their therapist, with others being less committed to or avoidant within the relationship. The final theme was about interpersonal support from others experiencing similar difficulties. Service users varied as to what level of support they would find useful, ranging from accessing previously recorded stories and suggestions to more active involvement in digital or face-to-face support groups.

#### Define

The define phase involved defining the design brief based on the insights from the discover work. A number of possibilities for the therapy redesign were identified, including family and carer involvement, social inclusion, peer support, and self-help. The areas of impact that appeared most relevant to improving usability were optimizing therapists’ and service users’ time within and between sessions and improving self-monitoring and self-management in daily life. The design brief was then generated by identifying the factors that could limit how useful the therapy was during and outside of therapy sessions (ie, the problem paradox). The design brief, therefore, specified that we aimed to develop a digital platform to support the therapy process by:

1. Supporting people to notice their thoughts and thinking habits
2. Presenting information in a simple and memorable way
3. Being enjoyable and trustworthy
4. Promoting personalization and normalization
5. Helping people feel more supported *and* independent.

#### Develop

Multimedia Appendix 3 illustrates the key processes and outputs during the develop phase, including concept generation, concept development, concept evaluation, narrative prototypes, modality prototypes, and participants’ feedback on the prototype testing. The develop phase commenced with creative workshops involving clinicians, industrial designers, and game developers. On the basis of the design brief, we generated concepts for optimizing each therapy session and the time between sessions. Sixty concepts were suggested, which were grouped by theme resulting in 11 overarching concepts. These were then subject to further concept development by detailing what the therapy could look like if it was designed according to the concept. The developed concepts were then rated according to ease of implementation, likely impact, and appeal. On the basis of these ratings, 3 concepts were selected for narrative prototype testing. These concepts were bubbles, where thoughts are visualized as bubbles that can be influenced by our actions; journey, where therapy is framed as an incremental process with challenges and achievements; and interaction, which focused on providing simple and habitual tools for dealing with worries.

The selected concepts were prototyped digitally and validated by presenting them to participants on a tablet. The validation process focused on both participants’ verbal reports and their behavior in relation to the prototypes. The concept of illustrating thoughts as bubbles resonated strongly. Participants displayed positive affect and approach behavior responses. Importantly, with regard to the aims of psychological therapy, the metaphor helped them see their thoughts as transient and separate from the self. They noted that bubbles could have different sizes depending on their intensity and that their movement, speed, and color could reflect different thinking patterns and styles. Participants also liked the idea of therapy represented as a journey, where each session is characterized by new experiences guided by their digital avatar who interacts with other characters along the way. The interaction prototype was less appealing to users who had a neutral or confused affective response and commented that it felt too abstract and oversimplified their problems. Bubbles and journey were therefore selected as the design concepts for framing the therapy redesign.

The second prototype testing explored the uptake and usability of different modalities for monitoring thoughts (text questions, camera, voice recorder, and counter) using a design probe. Participants were given a basic smartphone with the prototype installed for them to use over a week. They were told to use the prototype as they wished to explore if and how they engaged in using the smartphone to monitor their worries. At the end of the testing period, daily data indicated 87% (24/28) usage for the text questions, 50% usage for the voice recorder and counter (14/28), and 34% (10/28) for the camera. This suggested a preference for simple text as the main monitoring modality, although it was notable that the voice recorder and counter were also used, despite being considerably harder to access on the phone’s interface. In addition, participants reported a mean rating of 73% for enjoyment, 61% for usability, and 85% for acceptability on a 10-item User Experience Survey (adapted from [49]) designed specifically for the testing. Participants’ feedback showed that monitoring was viewed as valuable, enjoyable, and easier in digital modality than using pen and paper materials. Unsurprisingly, given the basic and unintuitive handset, participants noted the prototype was quite difficult to use. There were further concerns about privacy and impact on paranoia. All participants wanted more support from the phone to manage their worries.

#### Deliver

##### Wireframe Storyboard Development

In the deliver phase, wireframe storyboards of the session and out-of-session content were developed based on the selected concepts and then iteratively coded alongside user testing. All the session content from Thinking Well was incorporated, with a redesigned interface and functionality. An analogue aesthetic (ie, life-like illustration) was used throughout to provide an accessible and friendly design for people less willing and able to use technology. The use of written text was significantly reduced and replaced with short audio files or simple visual displays. Haptic interactions were used, where possible, to promote engagement, enjoyment, and memorability. The mobile app was designed so that people could use it without the keyboard if they wished, improving accessibility for those less digitally literate. The flow through the interface was designed to increase the likelihood of sustained engagement and completion of therapeutic tasks. For example, *next* buttons were made more visually salient than *back* or *exit* buttons so that users were more likely to tap them and sustain their engagement.

Rapid prototyping and testing also explored the aesthetic of the bubbles used to visualize thoughts and thinking habits, given that they represented a unifying visual language in the therapy. On the basis of the design research insights, a balance was sought between an appealing appearance that increased the likelihood of people wanting to use it and a wish not to invalidate their concerns. It was anticipated this would help people to see their thoughts as less threatening and separate from themselves. Visual attributes (eg, size, movement, and color) and ways of interacting with the bubbles (eg, scaling, tapping, moving, and popping) were investigated as a way of communicating information about the nature of thoughts and how we can relate to them. It was decided that the size of the bubble would reflect the intensity of the thought, whereas the speed at which it spins would illustrate the associated thinking habit. Worries are shown as gray bubbles, safer thoughts or other strategies for feeling safer are displayed as colored bubbles, and worries that the person has *slowed down* are given a colored halo. A finger tap was chosen for selecting a thought and its color, with scaling used to alter the bubble size or spinning speed.

The therapy name, SlowMo, was the product of a brainstorming workshop with designers, psychologists, and software developers. Workshop participants were given the aim of finding a name that would appeal to both service users and therapists, that communicated the essence of the therapy, that was phonetically engaging and memorable, and that could function within the clinical context (eg, when clinicians were referring service users or in therapy discharge reports). Popular digital brand names were reviewed for inspiration, and name concepts were generated based on the themes of *care and compassion*, *feeling safe and calm*, and *tools and superpowers*. Over 200 concepts were developed; each participant selected their favorites, which were then reviewed. SlowMo was selected, supported by the tagline *slow down for a moment*.

##### SlowMo: Minimal Viable Product

**Figure 2:**
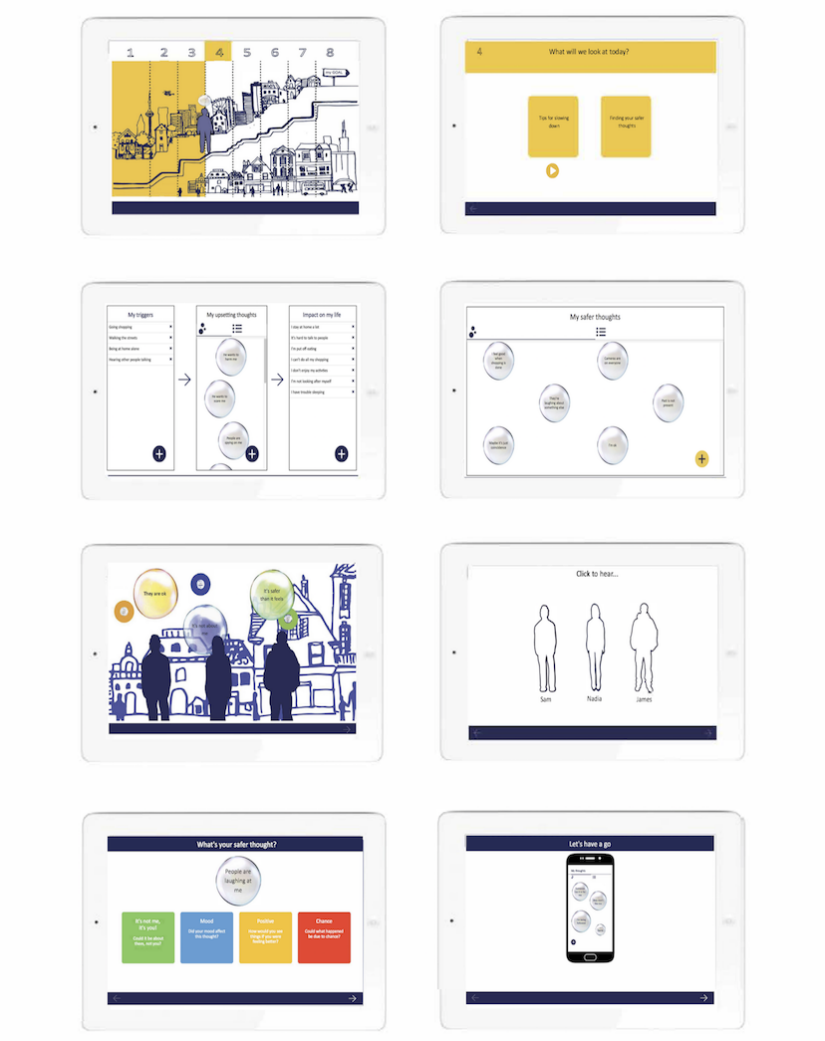
Main screens from the SlowMo Web app (from left to right, top to bottom): journey screen for navigating the sessions, aims screen, worries formulation, safer thoughts formulation, animated screen providing psychoeducation, avatar screen providing normalizing stories, example task for slowing down thoughts, and prompt screen for in-session practice of the app.

**Figure 3:**
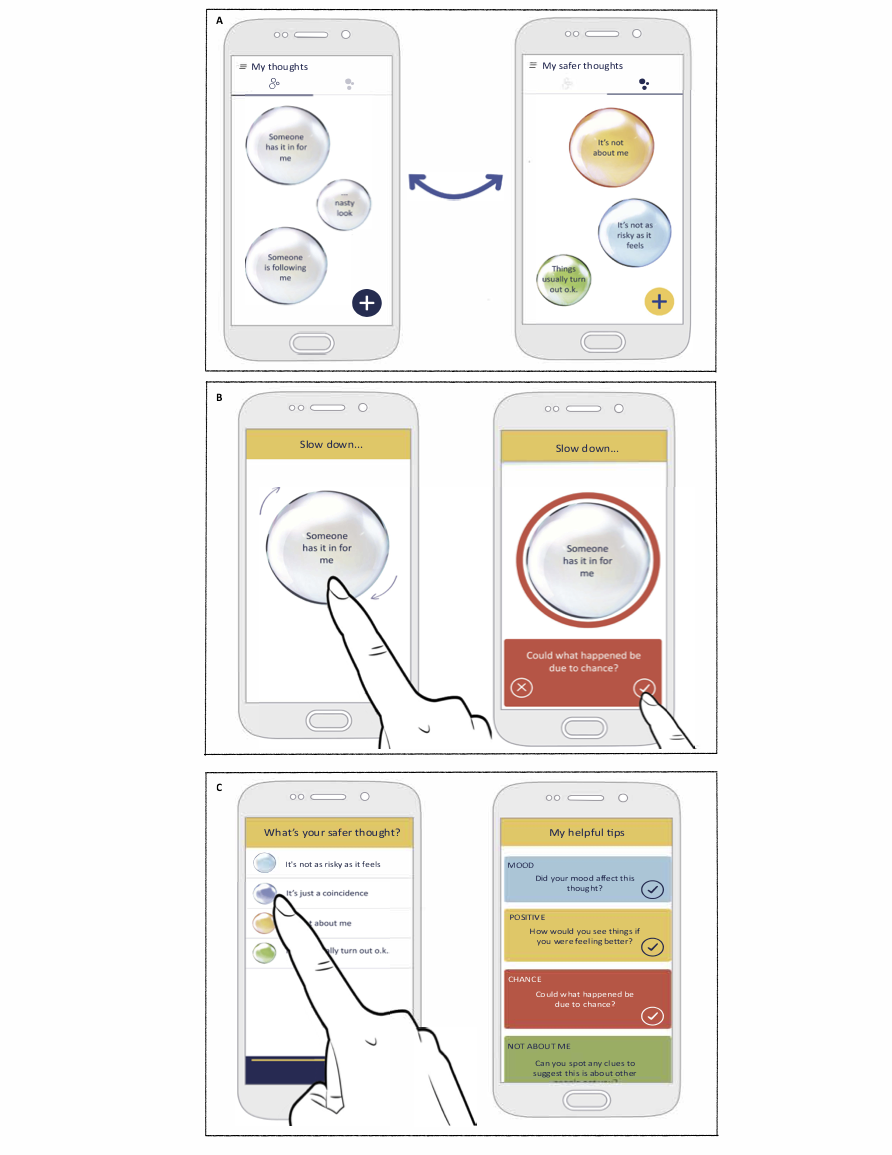
Main screens from the SlowMo mobile app: A) The home screen displays worries and safer thoughts; B) When experiencing a worry, the app encourages the user to slow down for a moment and provides tips to support finding safer, alternative thoughts; C) The app provides easy access to users' personalized safer thoughts and helpful tips.

The main screens from the SlowMo Web app and mobile app are shown in Figures 2 and 3, respectively, with further details provided in Multimedia Appendices 4 and 5. The wireframed storyboards were iteratively coded alongside user testing to produce the minimal viable product. SlowMo consists of 8 individual, face-to-face sessions, lasting 60 to 90 min, which are supported by a Web app delivered on a laptop or tablet. When a person starts therapy, a unique user profile is set up, which is linked to an identification code. No personally identifiable information is required by the system. The identification code allows the user-entered data to be stored on the Web app, which are then synchronized during sessions to a native, android app for use in daily life. It was decided to use the identification code and native app as a way of minimizing concerns about privacy and security. People may also choose to not link the app to their user profile so that no data are transferred. Another advantage of the native app is that it minimizes financial costs as no internet connection is required, ensuring it has minimal provider costs and is thus accessible to low-income users.

##### SlowMo Web App: Minimal Viable Product

The Web app has a fixed structure to support fidelity and adherence, although content can be skipped to allow tailoring of the material to the person’s cognitive needs. The journey concept is used to anchor the therapy. During set-up, people select an avatar to represent them on their therapy journey and input a chosen name. The home screen then displays the person’s journey through therapy from which individual sessions can be accessed. The journey home screen also contains a destination signpost where people enter their personal valued goal for the therapy. As with previous versions of the therapy, initial sessions involve building the meta-cognitive skill of noticing thoughts and thinking habits. People learn that although fast thinking is common and can be useful, slow thinking can be helpful in dealing with stress and worries about other people. This principle is expanded in subsequent sessions by covering a new topic area and a related *slow down for a moment* tip. The topics from sessions 1 to 8 are notice your thoughts; notice your thinking habits; slow down for a moment; slow down: what is your safer thought; use a safety strategy; slow down: past experiences; slow down: pop the worry; and making a habit of slowing down. With some exceptions in sessions 1 and 8 for initial and final tasks, sessions follow a consistent format of monitoring progress, reviewing the formulation (ie, an overview of triggers, worries, impact of worries, and alternative safer thoughts), collaborative agreement of session aims, psychoeducation, normalization, experiential tasks to personalize learning, recording of key learning, practice with the SlowMo native app, and documenting a goal for the week.

The interface for these tasks was developed in line with the design brief. Progress is monitored through scaling the visual appearance of bubbles (size for intensity, spinning speed for thinking habit, and transparency for conviction) to be more appealing and reduce the reliance on numerical rating and graphs. A formulation of people’s difficulties detailing triggers, worries, impact on life, and helpful thoughts and strategies is developed in session 1 using the visual language of bubbles. This is pulled through to remaining sessions and can be easily updated as new insights, difficulties, and ways of coping emerge. The potential aims are communicated through interactive boxes that are tapped to reveal their content to be more engaging and memorable and provide a shared understanding of the session structure. Psychoeducation information is presented with brief audio messages paired with illustrative animations. Three characters with prototypical experiences of paranoia share their stories as the therapy progresses. Their function is to provide normalizing messages about fears of harm from others (eg, that they are common in the general population) and to model how the SlowMo tips can be used to make sense of worries and feel safer. Experiential learning tasks are designed to optimize personalization and implementation in daily life, for example, by exploring the impact of fast thinking on daily life or by practicing the application of the SlowMo tips to worries selected from the formulation. Text or audio recordings are then made of the most important learning points from the session and of a note outlining a goal or key message for the week to support the person in making use of the therapy strategies. The learning messages are pulled through to the final session to support a personalized review of the therapy, from which the individual can choose a customized selection of the SlowMo tips for use after the end of therapy.

##### SlowMo Native App: Minimal Viable Product

There is an emphasis throughout the intervention on practicing skills inside and outside sessions, with the SlowMo native app providing a bridge between the therapy meetings and everyday life. The app works by unlocking new content toward the end of each session, based on the learning topic covered in the Web app. This new content is reviewed in session, and where possible, the therapist supports the person to practice the use of the app outside the consulting room. The home screen has 2 viewing modes, one displaying worries and the other *feeling safer* thoughts and strategies. In the first session, the home screens automatically populate from the data inputted to the Web app formulation, which consists of the person’s worry bubbles. People are then encouraged to use the app to identify their worries over the next week. When they experience a worry, they tap the associated bubble to record its occurrence and then size the bubble to indicate how distressing it is. An additional screen is unlocked on the app during session 2, where the thinking habit associated with the worry is rated by spinning the bubble faster or slower. From session 3, a *slow down* screen is added to the process, which displays a spinning bubble. This slows down when tapped, to act as a cue to *slow down for a moment* to manage worries. From session 4 onward, additional strategy prompts or tips are provided on this screen based on the topic covered in the session. When a user selects a tip, a halo corresponding to the tip color appears around the gray worry bubble, providing visual feedback that a helpful slowing down idea has been identified. Following the slow down screen, there is an option to record useful new information by way of audio or text and then select an alternative safer thought or strategy. The user finally rerates the distress associated with the worry to evaluate the impact of slowing down.

Data are stored in a format whereby, when experiencing recurrent concerns, people can readily access what was previously helpful. When a worry is tapped on the home screen, this will initially access a *thought profile* page from which users can either enter the slowing down process or see a summary of previous occasions when they have slowed the thought down (ie, the selected tip, information recording, safer thoughts, and pre- and postdistress rating). Another option is to access a list of all the tips that have been liked in relation to the thought. In addition, the burger menu of the app sequentially unlocks a brief summary of each session (under a *My journey* option) to act as an aide memoire for session content (ie, the slowing down tip, the message to self, the most important learning point, and monitoring ratings). The burger menu also consists of *settings*, where the offline mode can be selected, and at the end of therapy, an option is unlocked to allow the selection of slowing down tips. The burger menu also contains an *About SlowMo* section that briefly details the background to the development of SlowMo and privacy and security information. A *My safety plan* section advises users what to do in a crisis and provides an option to insert crisis contact numbers. Finally, optional notifications are available if people wish the app to provide prompts to encourage slowing down.

##### Technology Platform

The software development work was done by Evolyst Ltd, a user-centered and evidence-based health care software development company, informed by the British Standards Institute quality criteria and code of practice for health care apps [63]. SlowMo uses a proprietary software platform developed using an Azure-based Windows Communication Foundation Web Service, acting as an Application Programming Interface to a Model View Controller Asp.Net Web app, and a Xamarin.Android-based mobile app, allowing for use of the full Microsoft Stack and negating interoperability issues. SlowMo has currently been developed as a standalone product, given the lack of consensus on operating systems across the NHS trusts and current interoperability issues.

## Discussion

### Principal Findings

This study is the first to employ inclusive UCD methods within a design thinking approach to optimize the usability of an existing therapy for psychosis, Thinking Well. In the case series of a newly extended version of Thinking Well, we found indications of sustained medium to large effects on paranoia and well-being and small effects on reasoning post therapy and at 12-week follow-up. However, obstacles to the intervention interface were noted, underscoring the need for the design research. The inclusive UCD research identified the importance of therapy being usable, memorable, trustworthy, enjoyable, personalized, and normalizing, and of it offering flexible interpersonal support [27,37-39,42-44]. This led us to develop SlowMo, a blended digital therapy consisting of an intuitive Web app to augment the experience of face-to-face therapy sessions, which is synchronized with a native mobile app for use in daily life. By adding an app to the therapy, we hope to optimize its reach beyond the consulting room. SlowMo therapy is presented as a journey that supports people to notice the large, fast spinning, and gray worry bubbles that fuel distress and makes use of slow spinning and colored bubbles to shrink fears and feel safer. The use of personalization, ambient information, and visual metaphors provided a step change in therapy delivery to assist learning, monitoring, and management [36-37]. The application of inclusive UCD to the therapy interface may improve adherence, thereby increasing the likelihood of delivering benefit in real-world settings [64]. However, SlowMo requires further testing of its usability and usefulness. A feasibility study of the native app has been completed, with promising findings, while SlowMo’s overall effectiveness and the adherence and usage of both the Web app therapy sessions and the mobile app are currently being investigated in a multicenter, randomized controlled trial [23].

### Limitations and Future Directions

An important limitation of the study is the lack of integration of an implementation strategy within the therapy design. This is critical, given that most health technologies fail to be adopted, scaled-up, spread, and sustained, even where they are efficacious in randomized controlled trials [65]. The tailoring of the SlowMo design to its specific target problem, a range of intended users, and the delivery context may support initial adoption, together with the progress made in establishing its value proposition to stakeholders and technological requirements. However, even if SlowMo is found to be sufficiently usable and useful in our trial, there are significant challenges to it being embedded in health service care pathways across organizations, which will need to be tackled for successful implementation.

We therefore do not consider SlowMo to be a finished product, but rather a nascent behavioral intervention technology [66] or technology-enabled service [67]. The fundamental cognitive and behavioral principles of SlowMo will not change, given the theoretical underpinning and the robust findings from our previous empirical work [17]. However, we are developing the therapy interface iteratively, in the context of this trial, with the aim of moving toward a sustainable service. At this stage, we have funding for relatively minor and incremental changes. However, dependent on the trial outcomes, there are several target areas for further innovation, which may involve additional behavior change methods and technologies (eg, embodied conversational agents, online support groups, instant messaging, wearable biofeedback, and gamification) [68-71]. From an agile science perspective, SlowMo could be implemented as a module within a broader digital therapy platform for psychosis [42,72] or adapted for a range of other difficulties and settings. Its innovative redesign of a thought record, a widely used CBT tool, could be repurposed for other mental health difficulties. We are currently testing the feasibility of a stand-alone version of the app, Mo, to support stress management and well-being in the general population.

### Conclusions

In conclusion, this study is the first to demonstrate how an inclusive UCD method (which privileges the involvement of a wider range of service users than in conventional participatory design) can enhance the usability of therapy and augment developments in psychological theory and interventions. We hope that our study may serve as a prototypical example of how design thinking can challenge skeuomorphism in digital health, whereby therapy features made redundant by technology are unnecessarily replicated (eg, digitally replicating pen and paper tools such as thought records) instead of facilitating psychological mechanisms of change through innovative digital means. Notwithstanding the hugely valuable progress made over the past 2 decades in psychological therapy for psychosis [1], we echo recent calls to shift the frame of therapy radically to address the fundamental paradox that evidence-based psychological interventions are often not sufficiently helpful to bring about meaningful change [39,64,66,73]. We recommend the adoption of inclusive, UCD methods to develop novel digital solutions that embed psychological principles into daily life.

## Appendix

Multimedia Appendix 1 Screenshots of the Thinking Well therapy.

Multimedia Appendix 2 Design research processes and outputs from the discover phase.

Multimedia Appendix 3 Design research processes and outputs from the develop phase.

Multimedia Appendix 4 Screenshots of the SlowMo webapp from the deliver phase.

Multimedia Appendix 5 Screenshots of the SlowMo app from the deliver phase.

## Abbreviations

BNSS: Brief Negative Symptom Scale
CBT: cognitive behavioral therapy
CBTp: cognitive behavioral therapy for psychosis
GPTS: Green Paranoid Thoughts Scale
NHS: National Health Service
SAPS: Scale for the Assessment of Positive Symptoms
UCD: user-centered design
VAS: Visual Analog Scale
