# Supplementary material for: How Inclusive, User-Centered Design Research Can Improve Psychological Therapies for Psychosis: Development of SlowMo
Source: JMIR Ment Health. 2018 Dec 5;5(4):e11222. doi: 10.2196/11222 (PMC6300708; doi:10.2196/11222)
Supplement: Multimedia Appendix 1 [file mental_v5i4e11222_app1.pdf]

Example Screenshots of Thinking Well Therapy

Powerpoint slides from session one including images of the out-of-session paper thought record ('Making a Habit of Thinking Well sheet') and practice plan card ('Thinking Well Habit card').

What is Thinking Well?

- It is a 6 session talking therapy for people with upsetting thoughts about others
- It will involve learning steps to help you cope with your thoughts and get on with your life

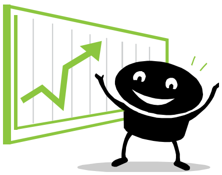

Module 1: Understanding Upsetting Thoughts

Aims of Meeting 1:

To find out:

- Upsetting thoughts about other people are common
- How our thinking habits affect upsetting thoughts
- How to notice your upsetting thoughts and start improving your thinking habits

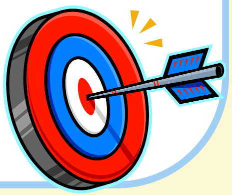

We all have upsetting thoughts about others…

Click on the boxes to listen to examples of when these people have had an upsetting thought about others:

'John'

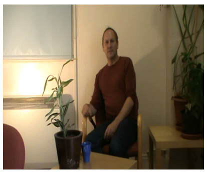

'Irene'

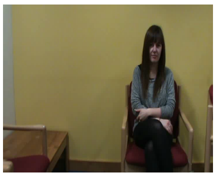

'Peter'

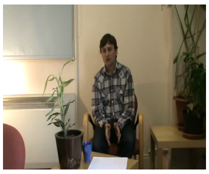

'Ben'

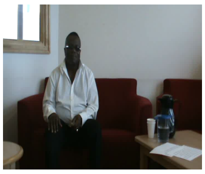

'Sandra'

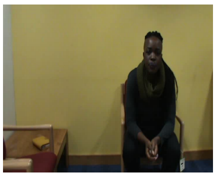

'Ali'

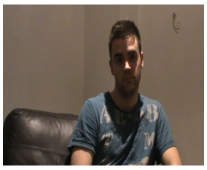

What triggers off my upsetting thoughts?

For example: feelings, noises, sensations, other people's behaviour…

Write down what triggers your thoughts on the *"Understanding my Upsetting Thoughts & Thinking Habits"* sheet

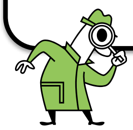

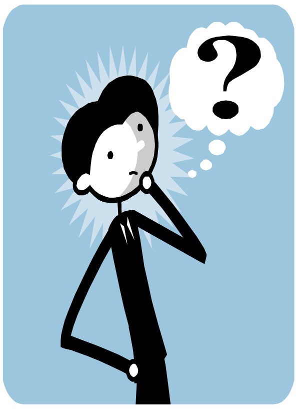

Making a habit of thinking well…

- Make a note of what happens when you use the step on the *Making a Habit of Thinking Well sheet*
- We can talk about what you noticed next time!

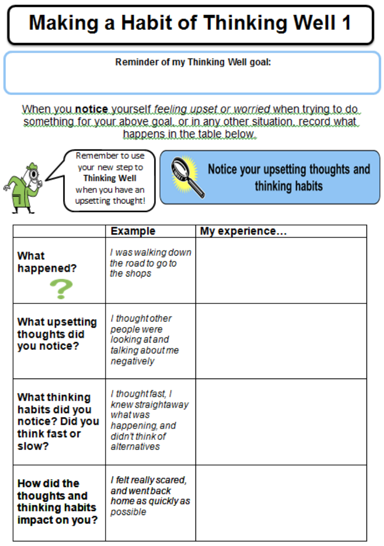

Making a Habit of Thinking Well 1

Reminder of my Thinking Well goal:

When you **notice** yourself *feeling upset or worried* when trying to do something for your above goal, or in any other situation, record what happens in the table below.

Remember to use your new step to **Thinking Well** when you have an upsetting thought!

Notice your upsetting thoughts and thinking habits

	Example	My experience…
**What happened?**	*I was walking down the road to go to the shops*	
**What upsetting thoughts did you notice?**	*I thought other people were looking at and talking about me negatively*	
**What thinking habits did you notice? Did you think fast or slow?**	*I thought fast, I knew straightaway what was happening, and didn't think of alternatives*	
**How did the thoughts and thinking habits impact on you?**	*I felt really scared, and went back home as quickly as possible*	

Module 1: Understanding Upsetting Thoughts

Making a habit of thinking well…

- It is really helpful to practise using the Thinking Well steps outside our meetings
  - When and where will you practise the step?
  - Could anything get in the way of you practising?
  - What will help you practice?
- Make a note of how you will use the step on the other side of your *Thinking Well Habit Card*

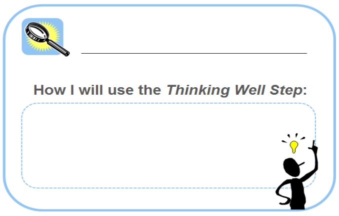

Key Points: Module 1

- Everyone has upsetting thoughts about others sometimes
- We have two main ways of thinking, or *thinking habits* – *thinking fast* and *thinking slow*
- When we *think fast*, we make up our minds quickly, go with our instincts and don't look for more evidence, and do not tend to change our minds
- *Thinking fast* can be helpful in some situations but it can mean we *'jump to conclusions'* and have an upsetting thought when we do not need to
- This programme will help you learn to improve your thinking habits so you can Think Well and feel better

My Weekly Ratings

Please answer the questions below about your worries over the past week:

How **distressing** has this been for you?

Not at all distressing 0 — 25 — 50 — 75 — 100 **Totally distressing**

How much have you **believed this is true?**

Believed not at all 0 — 25 — 50 — 75 — 100 **Believed absolutely**

Have you tried **to look for or to find out more information** about this?

Not at all 0 — 25 — 50 — 75 — 100 **All the time**

Have you thought it at all **possible that you were mistaken** about this?

Not at all 0 — 25 — 50 — 75 — 100 **Totally**

Have you thought of **any alternative explanations** for this?

Not at all 0 — 25 — 50 — 75 — 100 **All the time**

Example screenshots from the Thinking Well website for accessing therapy materials outside of sessions, including the homescreen, viewer for the session powerpoint slides, and digital thought record.

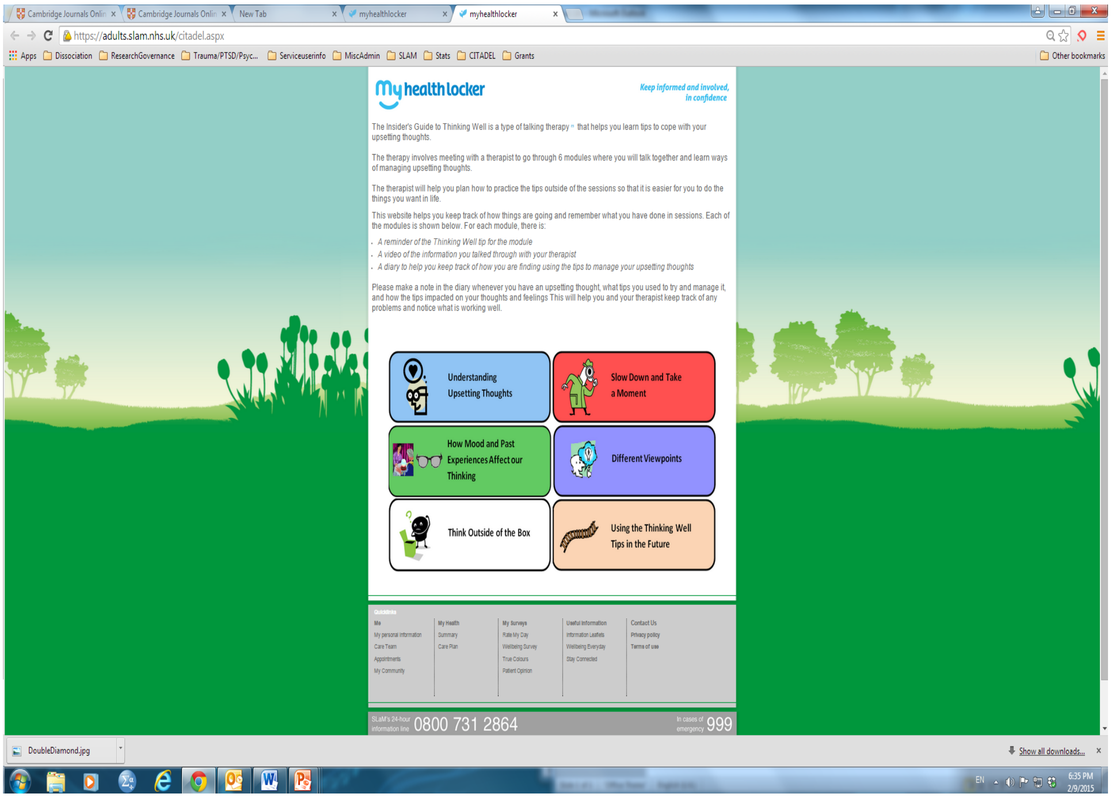

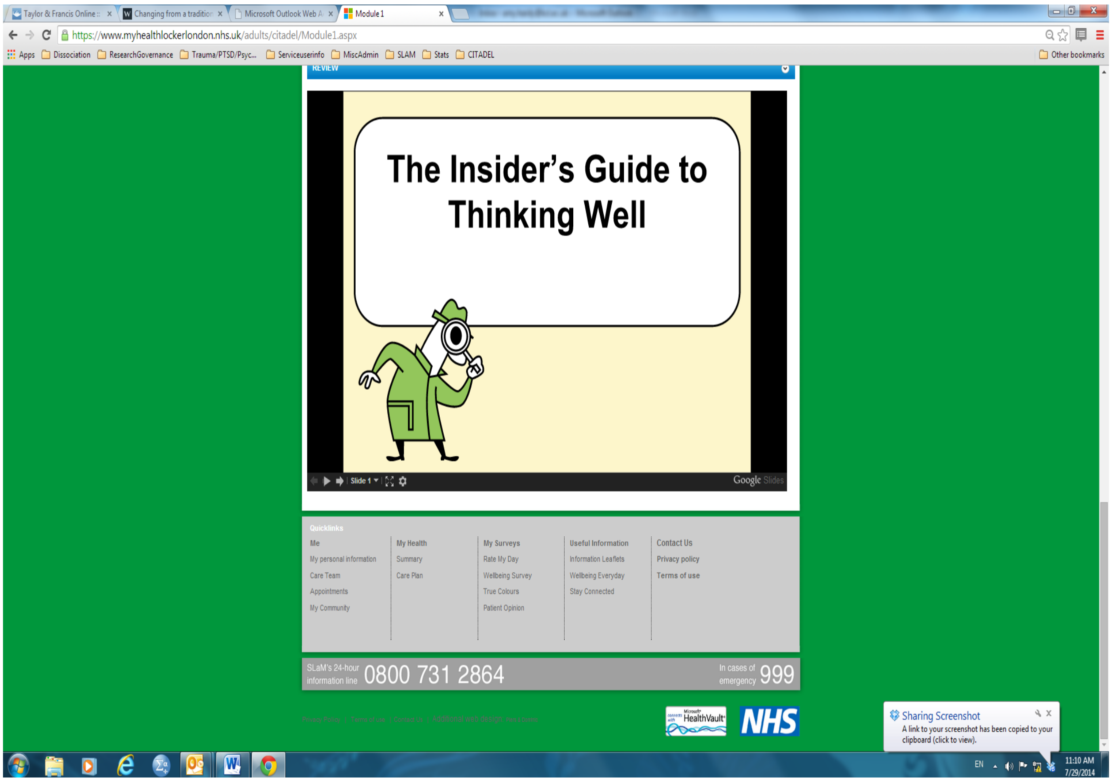

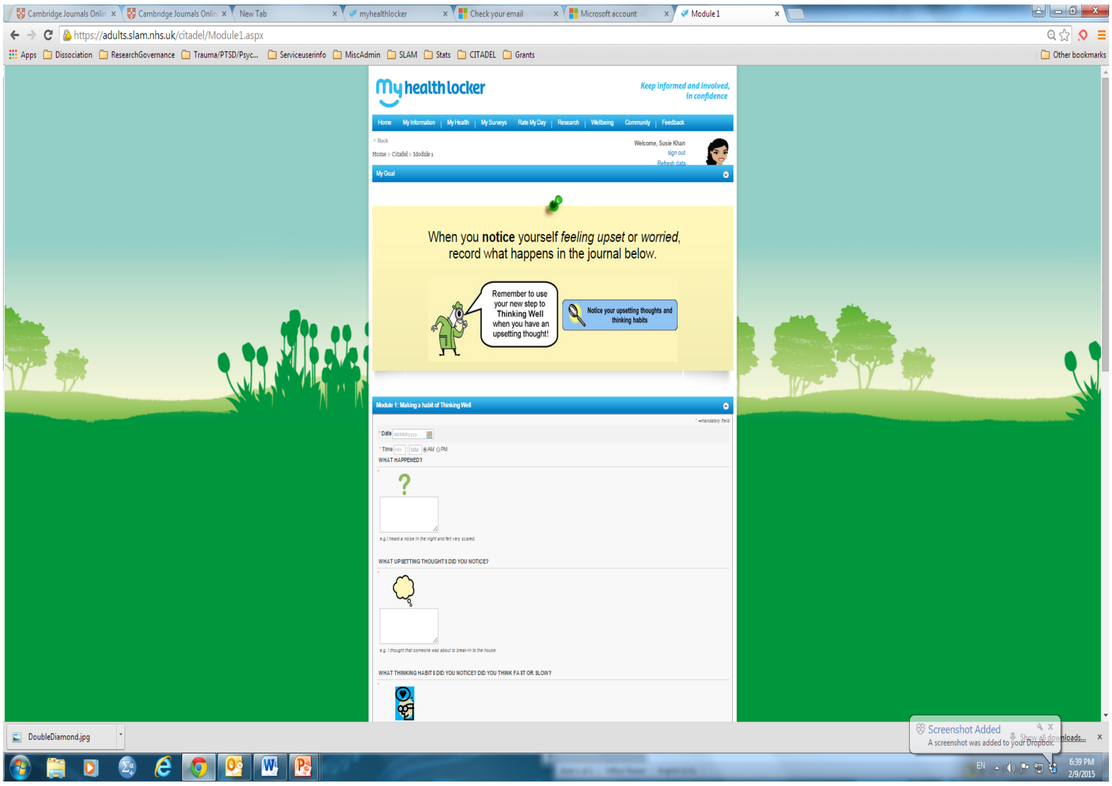

https://adults.slam.nhs.uk/citadel/Module1.aspx
My healthlocker
Keep informed and involved, in confidence
Welcome, Susie Khan
My Goal
When you notice yourself feeling upset or worried, record what happens in the journal below.
Remember to use your new step to Thinking Well when you have an upsetting thought!
Notice your upsetting thoughts and thinking habits
Module 1: Making a habit of Thinking Well
Date
Time
WHAT HAPPENED?
WHAT UPSETTING THOUGHTS DID YOU NOTICE?
WHAT THINKING HABITS DID YOU NOTICE? DID YOU THINK FAST OR SLOW?
Screenshot Added
A screenshot was added to your Dropbox.

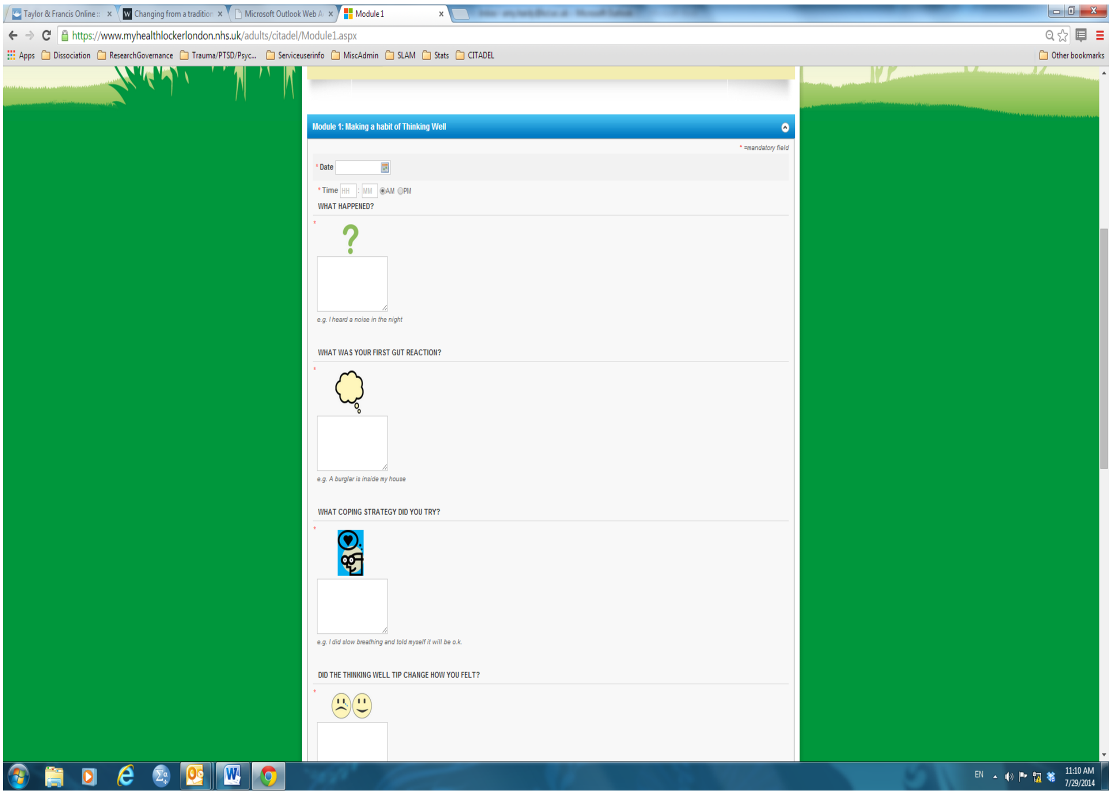

https://www.myhealthlockerlondon.nhs.uk/adults/citadel/Module1.aspx
Module 1: Making a habit of Thinking Well
* =mandatory field
Date
Time
WHAT HAPPENED?
e.g. I heard a noise in the night
WHAT WAS YOUR FIRST GUT REACTION?
e.g. A burglar is inside my house
WHAT COPING STRATEGY DID YOU TRY?
e.g. I did slow breathing and told myself it will be o.k.
DID THE THINKING WELL TIP CHANGE HOW YOU FELT?
