# Supplementary material for: How Inclusive, User-Centered Design Research Can Improve Psychological Therapies for Psychosis: Development of SlowMo
Source: JMIR Ment Health. 2018 Dec 5;5(4):e11222. doi: 10.2196/11222 (PMC6300708; doi:10.2196/11222)
Supplement: Multimedia Appendix 2 [file mental_v5i4e11222_app2.pdf]

Outputs from the *Discover* Phase of the Design Research

Process map of therapy sessions.

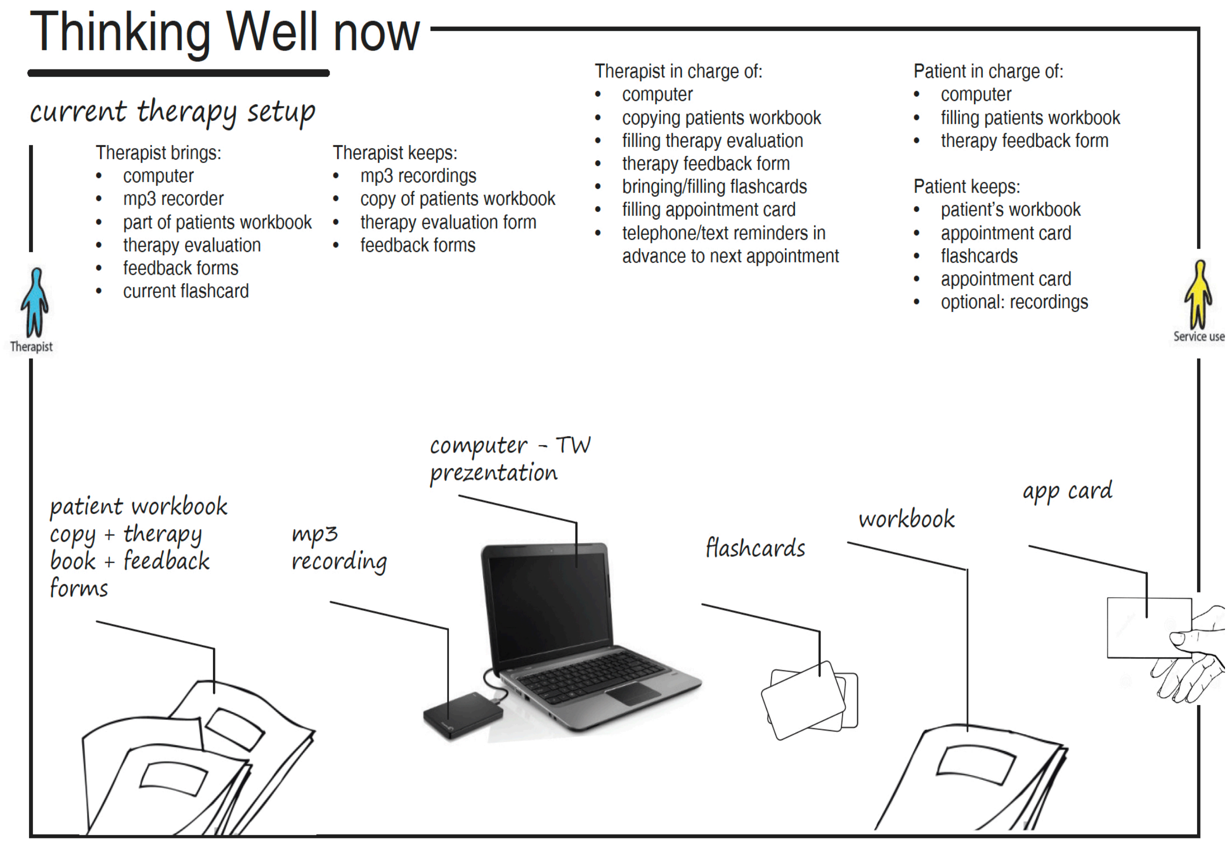

Process map of the service context.

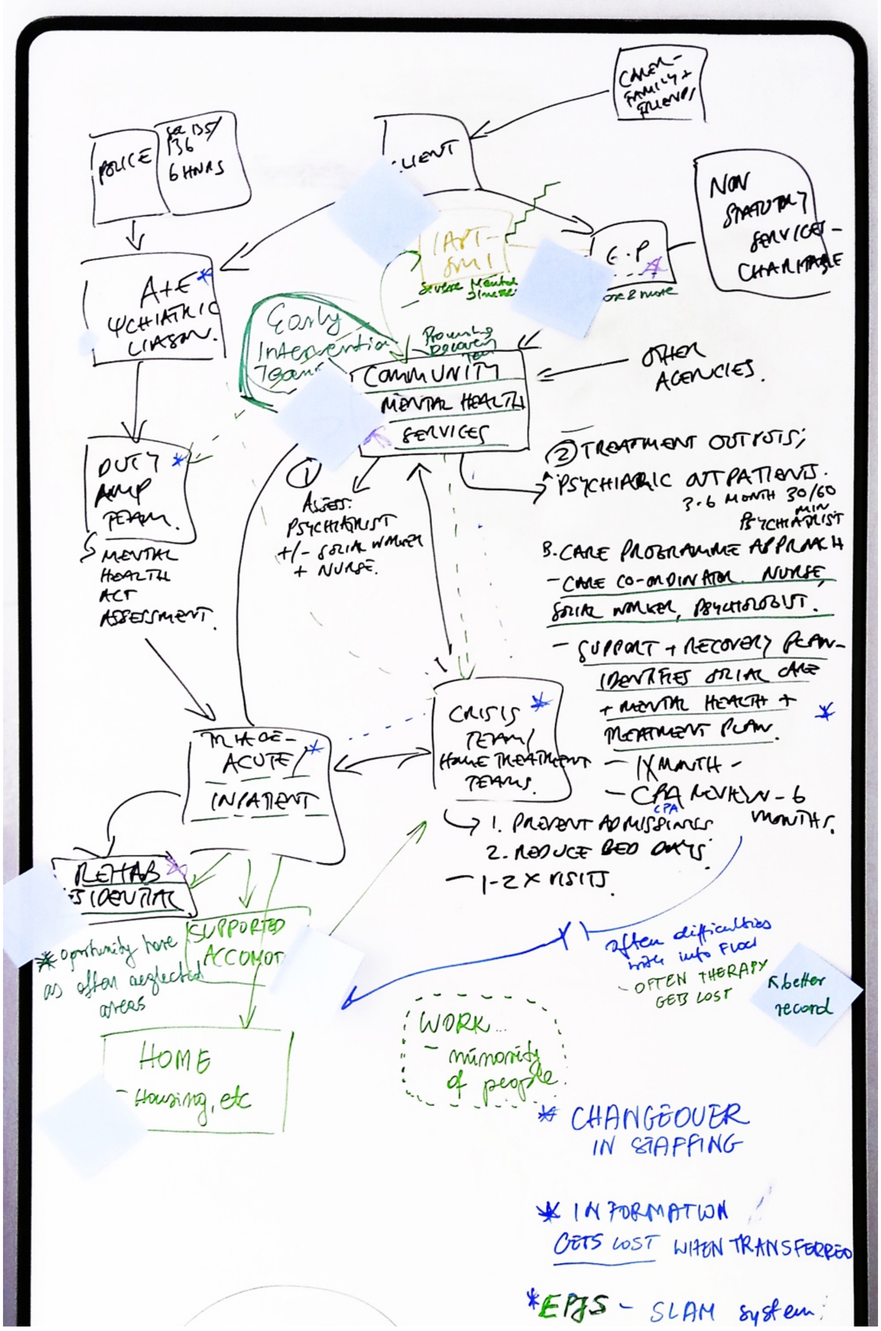

Service user journeys.

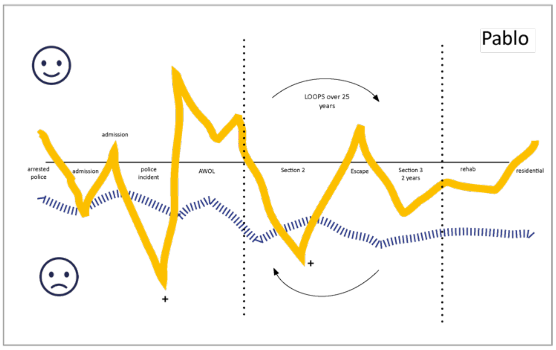

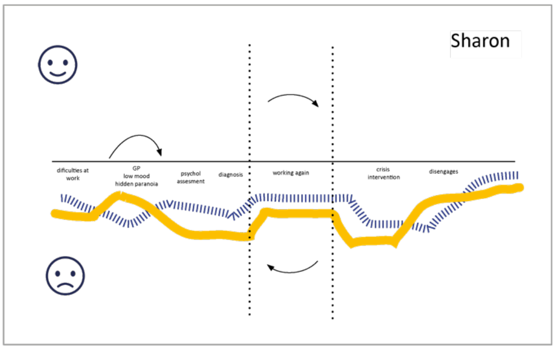

Service user profiles (with illustrative photos, not actual participants).

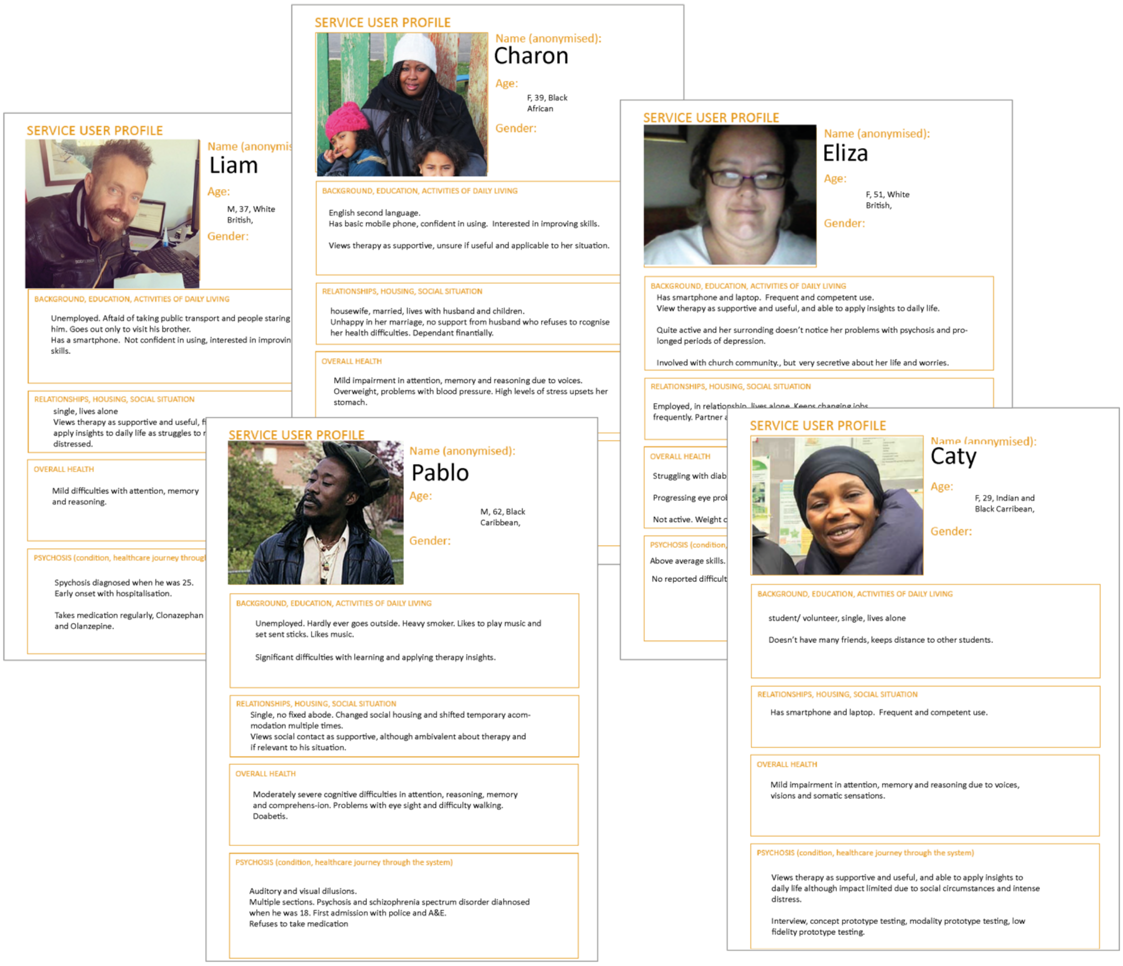

Moodboard for thoughts and emotions.

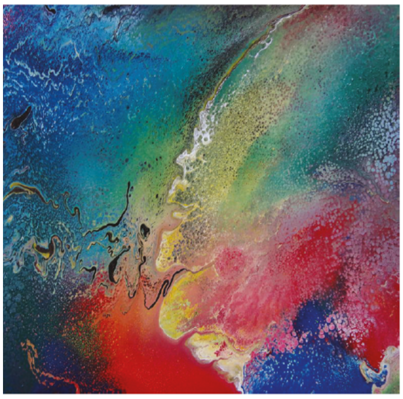
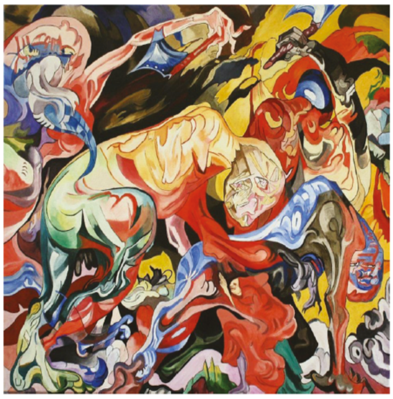
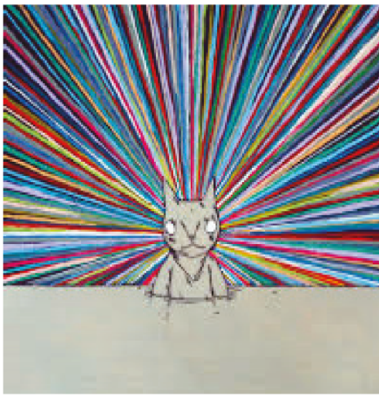
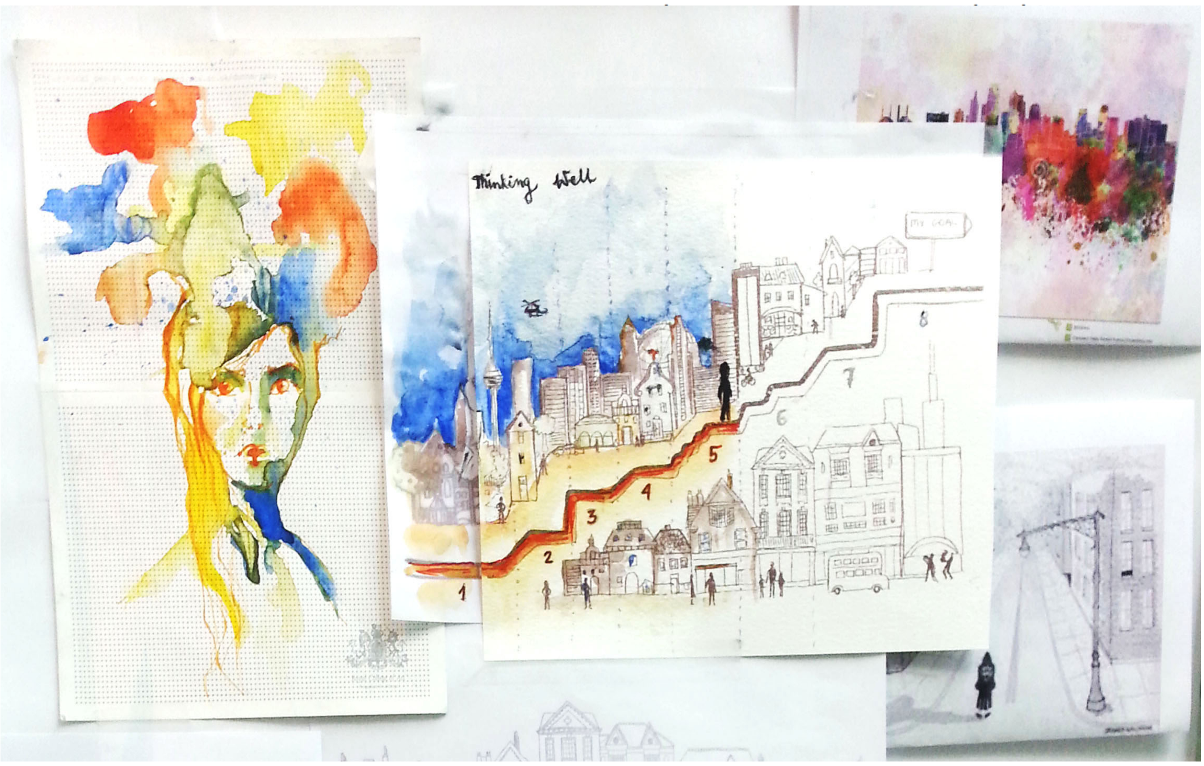

Themes emerging from the *discover* phase, with illustrative participant quotes.

Theme	Comments
Therapy usability	It was annoying to keep the notes. It would be much easier for me to listen to them than writing.<br>I almost forgot everything about therapy, I can't remember.<br>It was a lot of writing things down, too many questions.<br>I did not like the repetitiveness, there was a lot to take in and answer.<br>I made pictures of some therapy tips on my phone, so I can look at them when I'm stressed.<br>I keep the flashcards in my wallet.<br>I liked the fact my therapist made it personal to me, encouraging me to listen to my music and use lyrics to help me cope.<br>I really like the little cards. I take photos of them on my phone.
Technology	I've got my laptop, but I don't use it, I can't do it, I don't know.<br>I have problems with text on my phone, I can read it, but I can't text out.<br>I'm not sure about using technology in therapy. I'd worry about GPS and people tracking me.<br>I wish I could record reminders for myself during the session that later would be texted to me.<br>I would like to have a device I carry with me that would show me my progress.<br>I would love the therapy to be accessible on a phone, tablet or online.

	I'd like to have a wristband connected to the therapy to give me prompts, but it would have to be discrete and look good.<br>I'd like an app to give me different suggestions throughout the day, I would like it to be personalised to me.<br>Having a laptop opened up a new world for me. It helps me monitor myself. It would be useful if the therapy could be customised. Therapy helped build my confidence in using a computer.<br>I would like to be able to see how I've improved on a graph.<br>I'd like to be able to monitor my stress levels to see my progress and what I've achieved.
Enjoyment	I really enjoyed the videos during sessions.<br>I liked the exercises and games. It was good to know my results.<br>What I enjoyed most was the little games and tasks. It would be good to look at them again.<br>I liked the focus on practical skills and tasks.
Relationship to therapist	I do not want to share too much in or about therapy. I want to have control over what people know.<br>I don't want anyone else to know about therapy, I'd rather not have it every week.<br>The most important thing about therapy was that I felt like I'm being helped.<br>I'd prefer prompts to be a 2-way messaging service.

	The important thing about therapy was talking to the therapist and getting feedback, having somebody to talk to.
Interpersonal support	It was really helpful to know that other people are going through the same thing. Good to hear about their experiences and how they cope.
	I would love to belong to a support group. Somebody would have to look after the group, to make sure everyone had a voice.
	It would be helpful to have a virtual support group to connect all together; an expert could be there as well.
	I find it helpful to talk to people. It's helpful to hear about others' experiences.
	Sometimes its really difficult to come up with alternative ideas, it stressed me out, it would be great to see suggestions of what other people wrote.
