# Supplementary material for: How Inclusive, User-Centered Design Research Can Improve Psychological Therapies for Psychosis: Development of SlowMo
Source: JMIR Ment Health. 2018 Dec 5;5(4):e11222. doi: 10.2196/11222 (PMC6300708; doi:10.2196/11222)
Supplement: Multimedia Appendix 3 [file mental_v5i4e11222_app3.pdf]

Outputs from the *Develop* Phase of the Design Research

Concept generation.

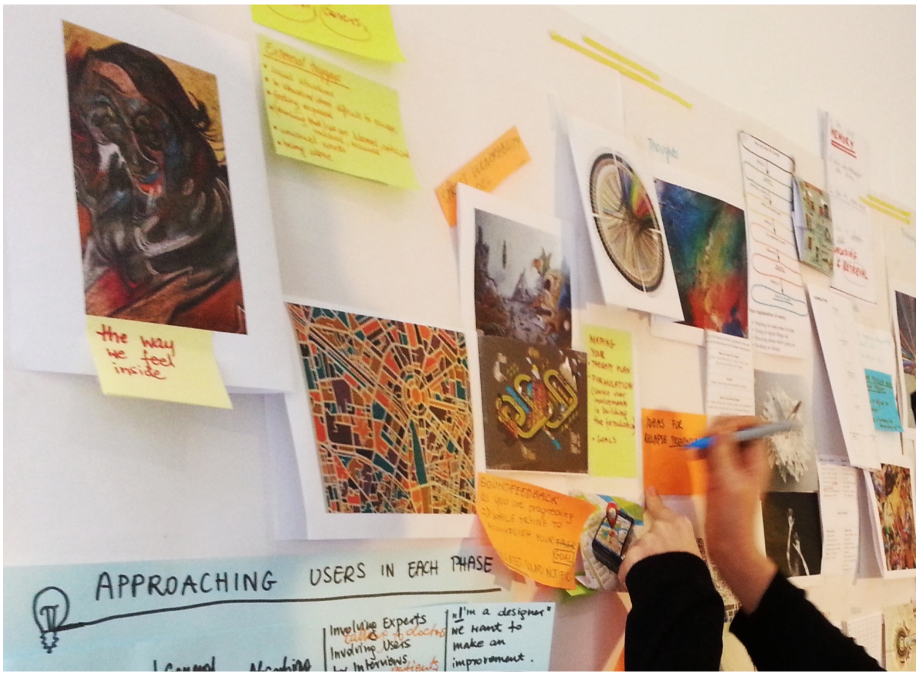

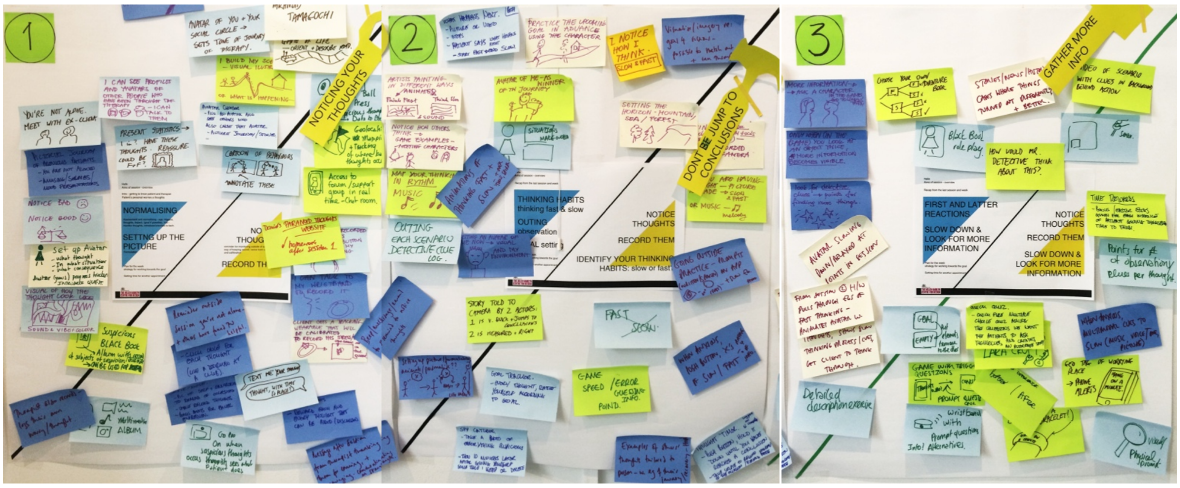

Concept development.

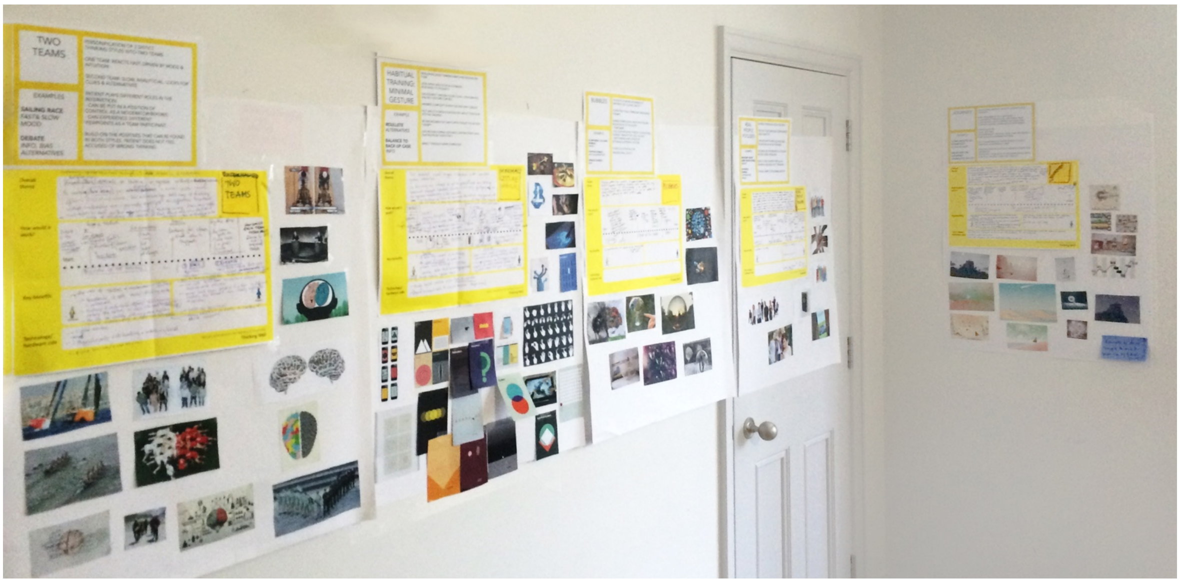

Concept evaluation.

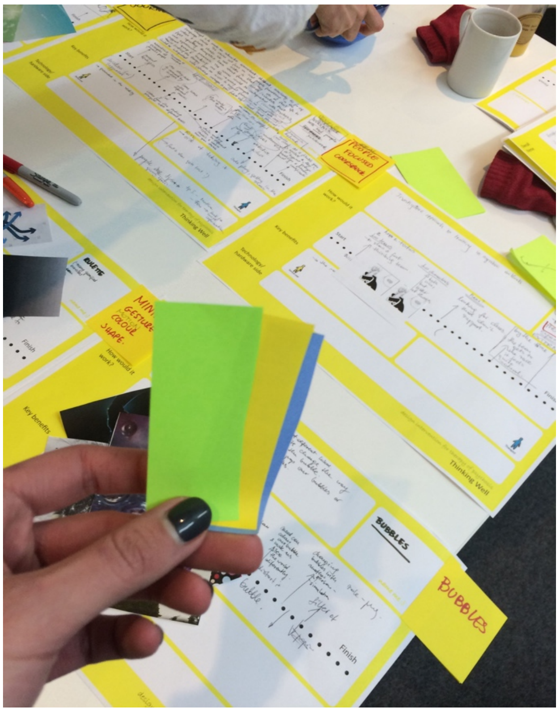

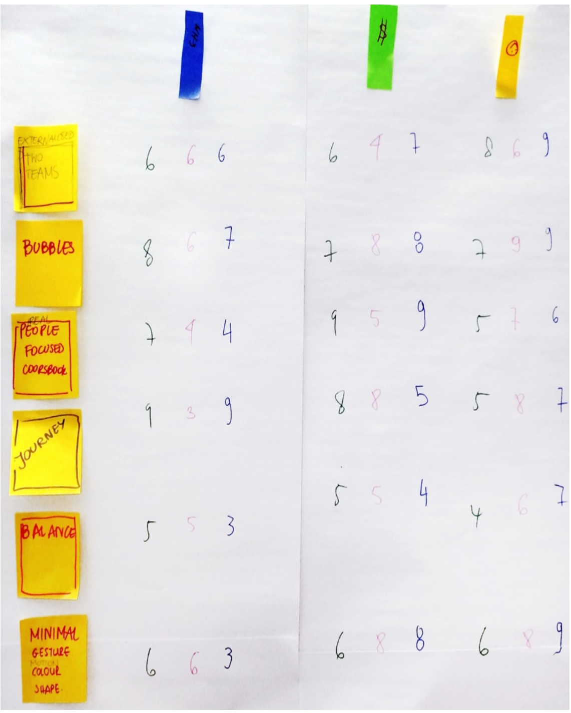

Narrative prototype testing.

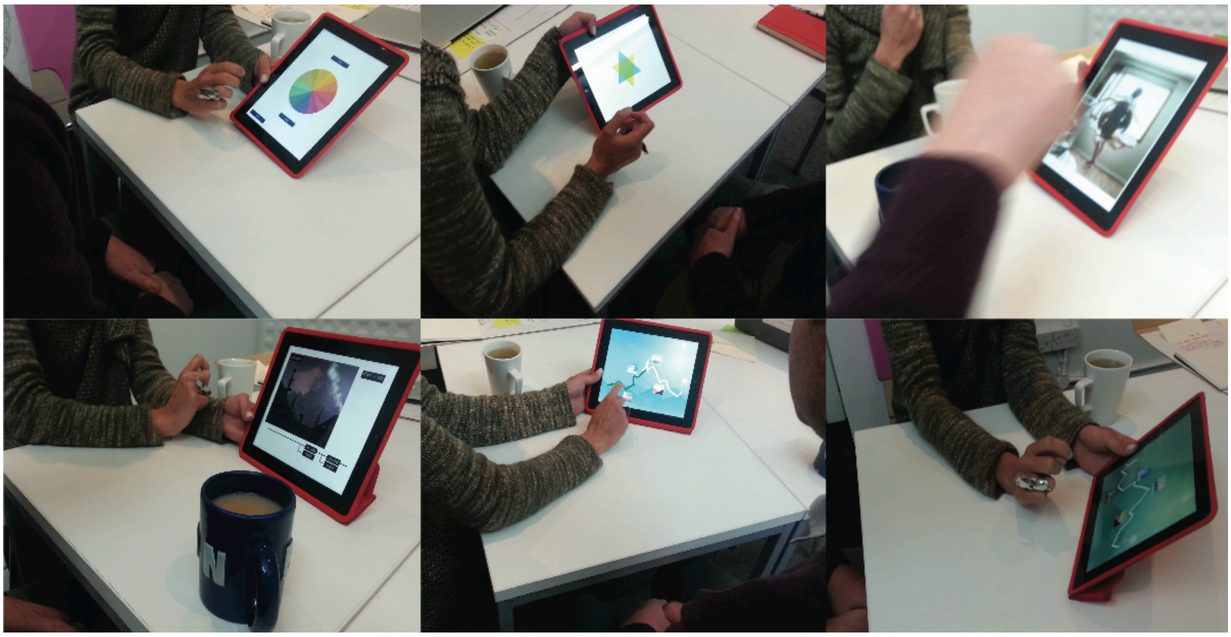

Modality prototype testing.

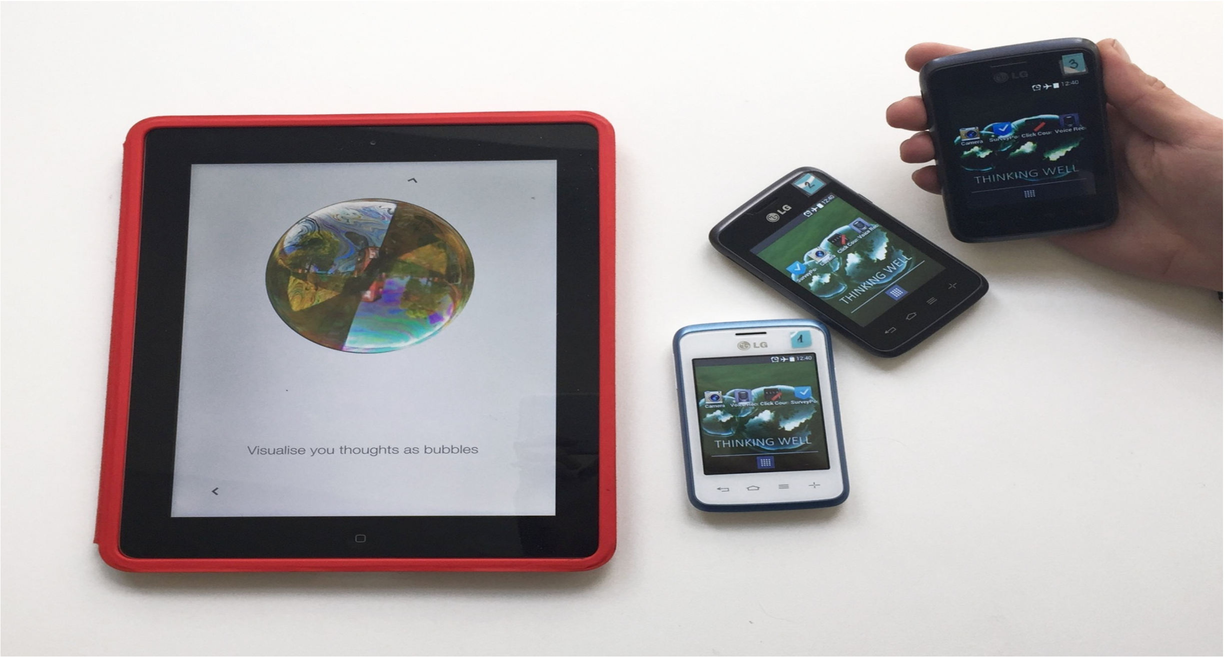

Modality monitoring prototype feedback.

Theme	Comments
Enjoyment	I liked doing it, good to have something to do.<br><br>It was good because it felt like people were keeping an eye on me and were being protective.<br>I really enjoyed it. Really liked the survey and recorder. Found the recording much easier to just say how I felt.
Acceptability	The phone helped me to get my upsetting thoughts out of my head and distract me.<br>I felt better after entering things into the phone about feeling stressed.<br>Sometimes it felt a bit much, needed a bit of space from it but got more used to it.<br>I worried about how secure it was. All the apps were good, voice recorder probably the best.<br>It helped listening to recordings to recognise what is difficult and feel like thoughts are being acknowledged.<br>I wanted to record generally how things were going, have a survey that captures positive as well as negative thoughts.
Usability	It was easy to do. I'm not good with phones but it was all right.<br>I preferred it to writing things down on paper. The phone was 1000x better than having to write things down. The apps are short and to the point. I can answer without having to think too hard.<br>The voice recorder recorded me when I didn't ask it too – not sure if I pressed record by mistake. I got very, very paranoid as a result.<br>The survey didn't work which was very frustrating.<br><br>It's harder to use the phone when feeling low.
