# Supplementary material for: How Inclusive, User-Centered Design Research Can Improve Psychological Therapies for Psychosis: Development of SlowMo
Source: JMIR Ment Health. 2018 Dec 5;5(4):e11222. doi: 10.2196/11222 (PMC6300708; doi:10.2196/11222)

The SlowMo webapp journey home screen displays the user's progress through therapy, and is used to access therapy sessions, the therapy goal and formulation.

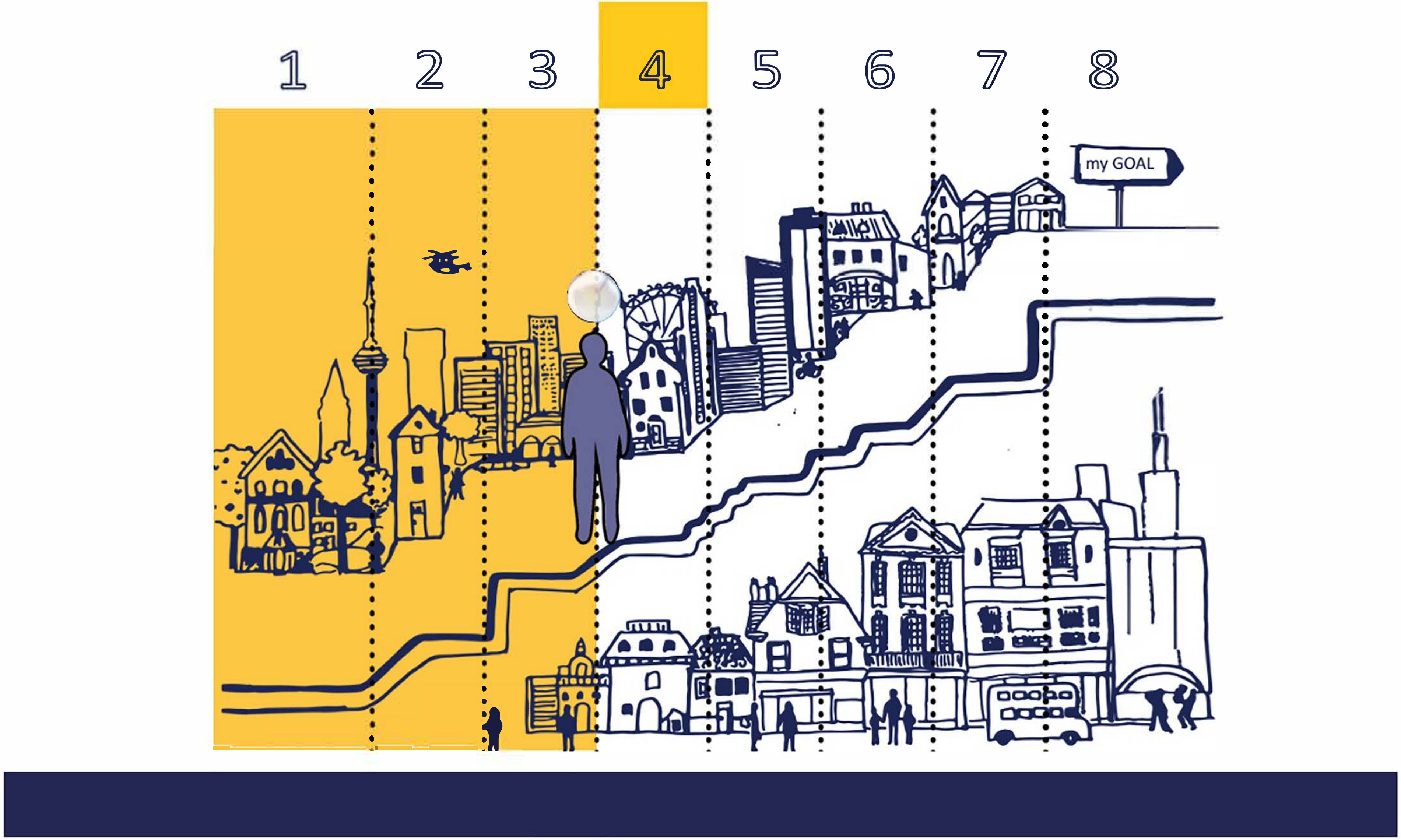

The personalised welcome page is shown at the beginning of each session, with the user's chosen name and avatar.

1

Hi James!

Welcome to session 1.

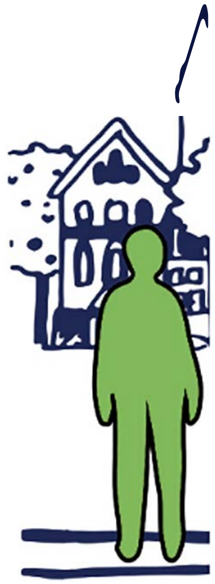

The formulation of worries, including their content, triggers and impact, is populated during session 1, then reviewed and updated at the start of subsequent sessions.

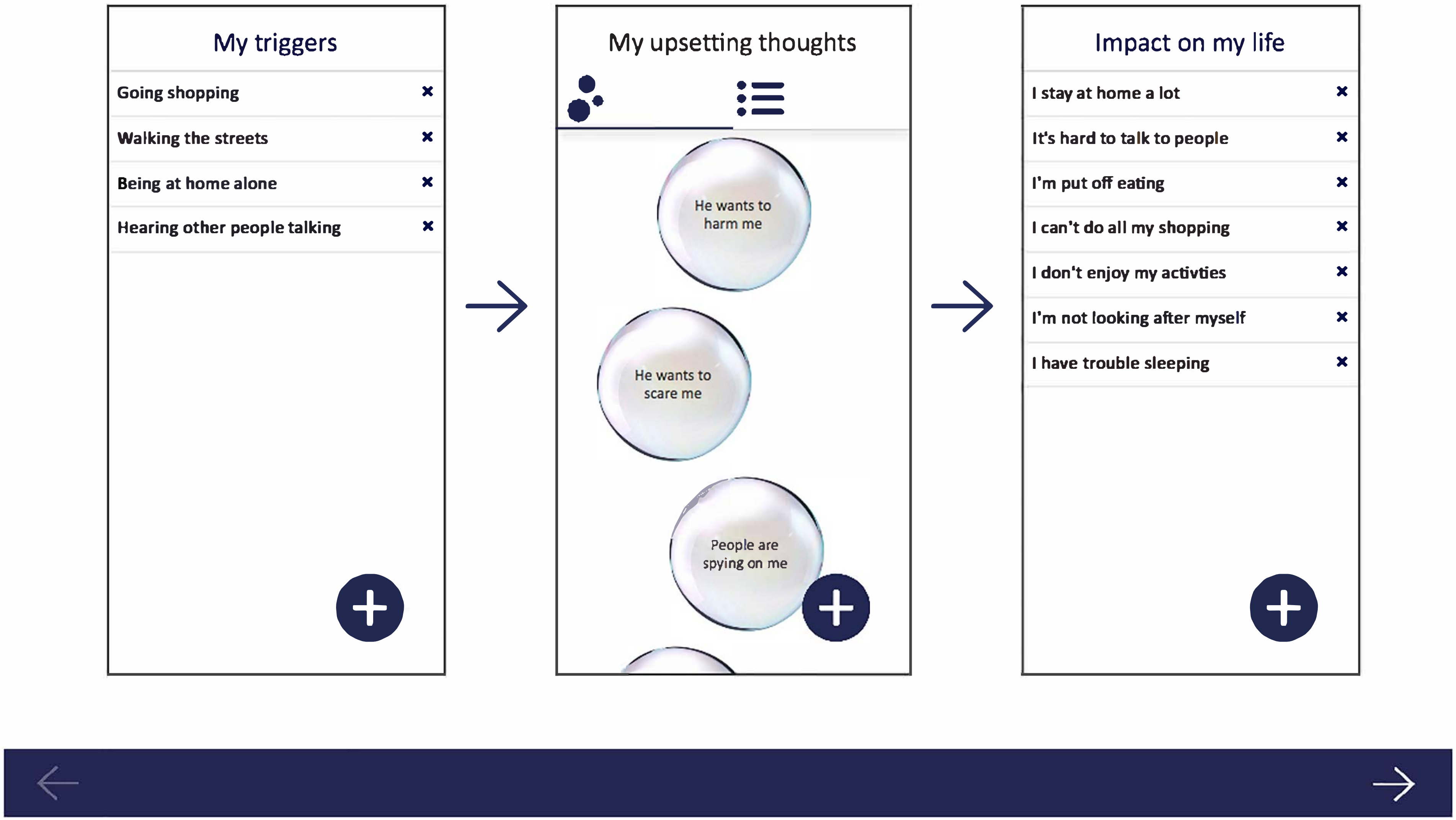

The formulation of safer thoughts is started in session 1,
then reviewed and updated at the start of subsequent sessions.

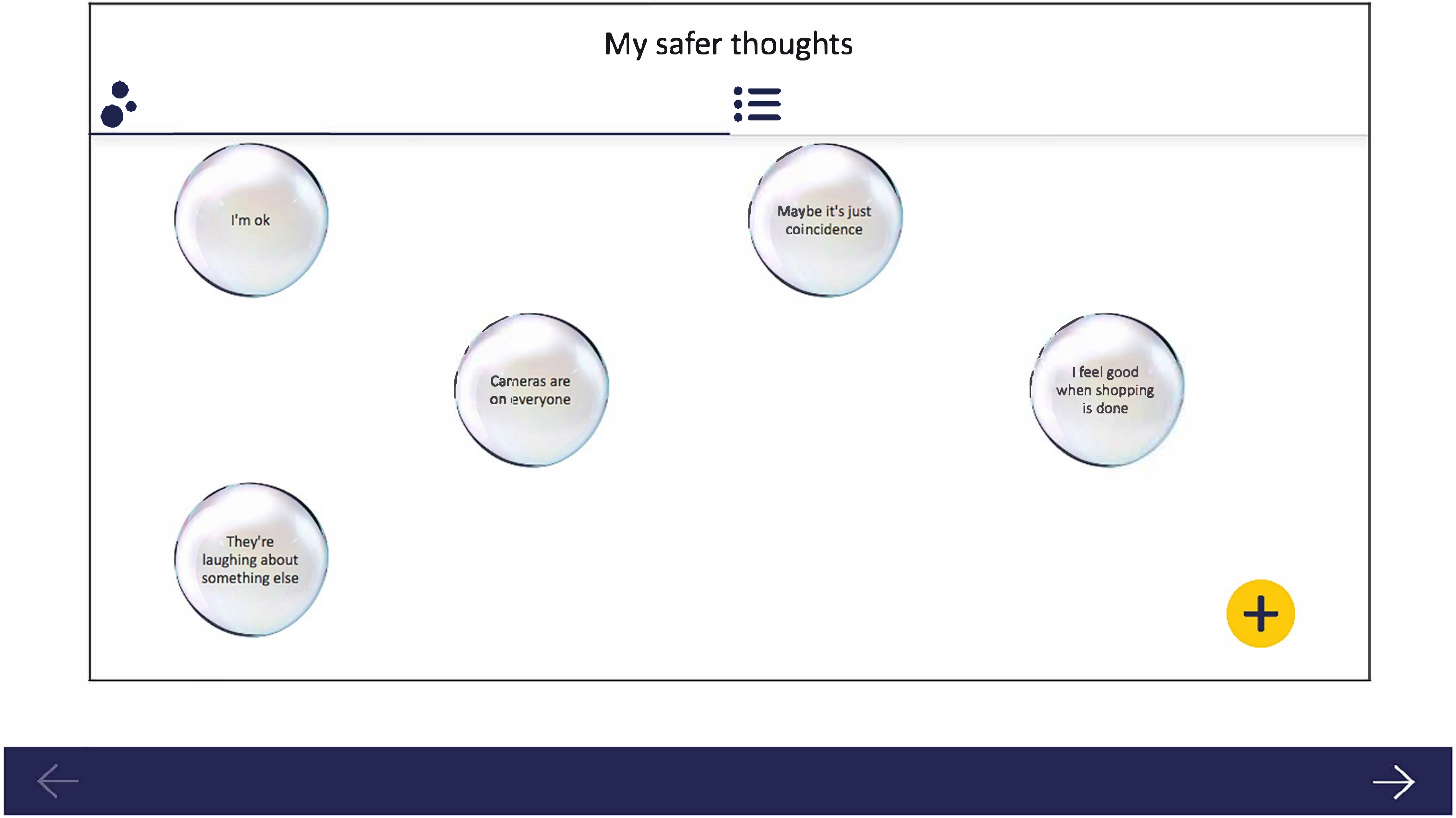

Each week users rate distress, conviction and thinking habits in relation to their worries.

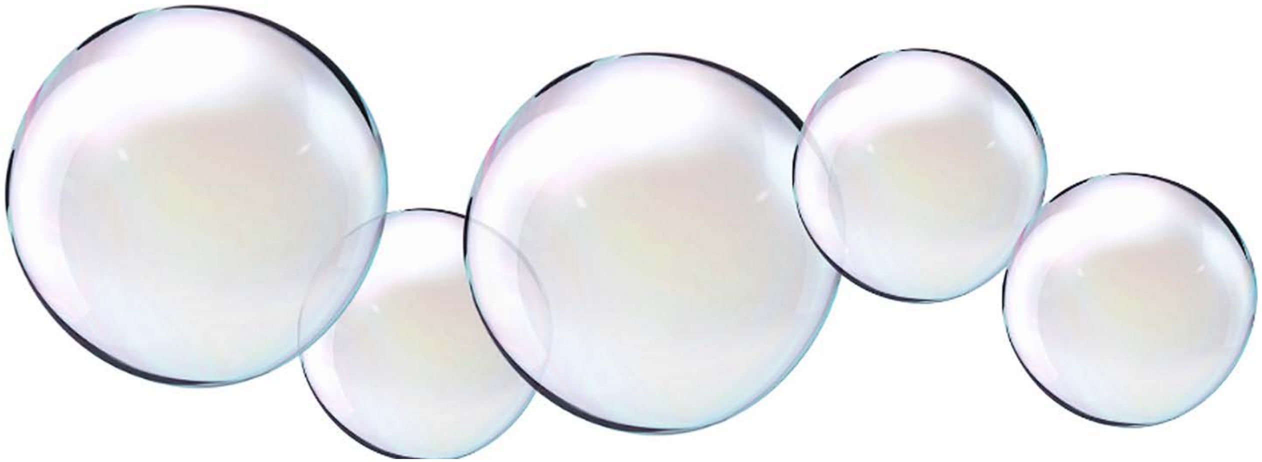

How distressing?

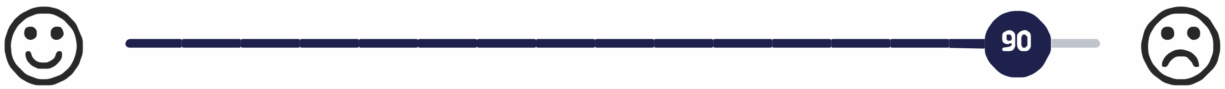

The aims screen displays boxes which the user taps to access an overview of the topics for the session.

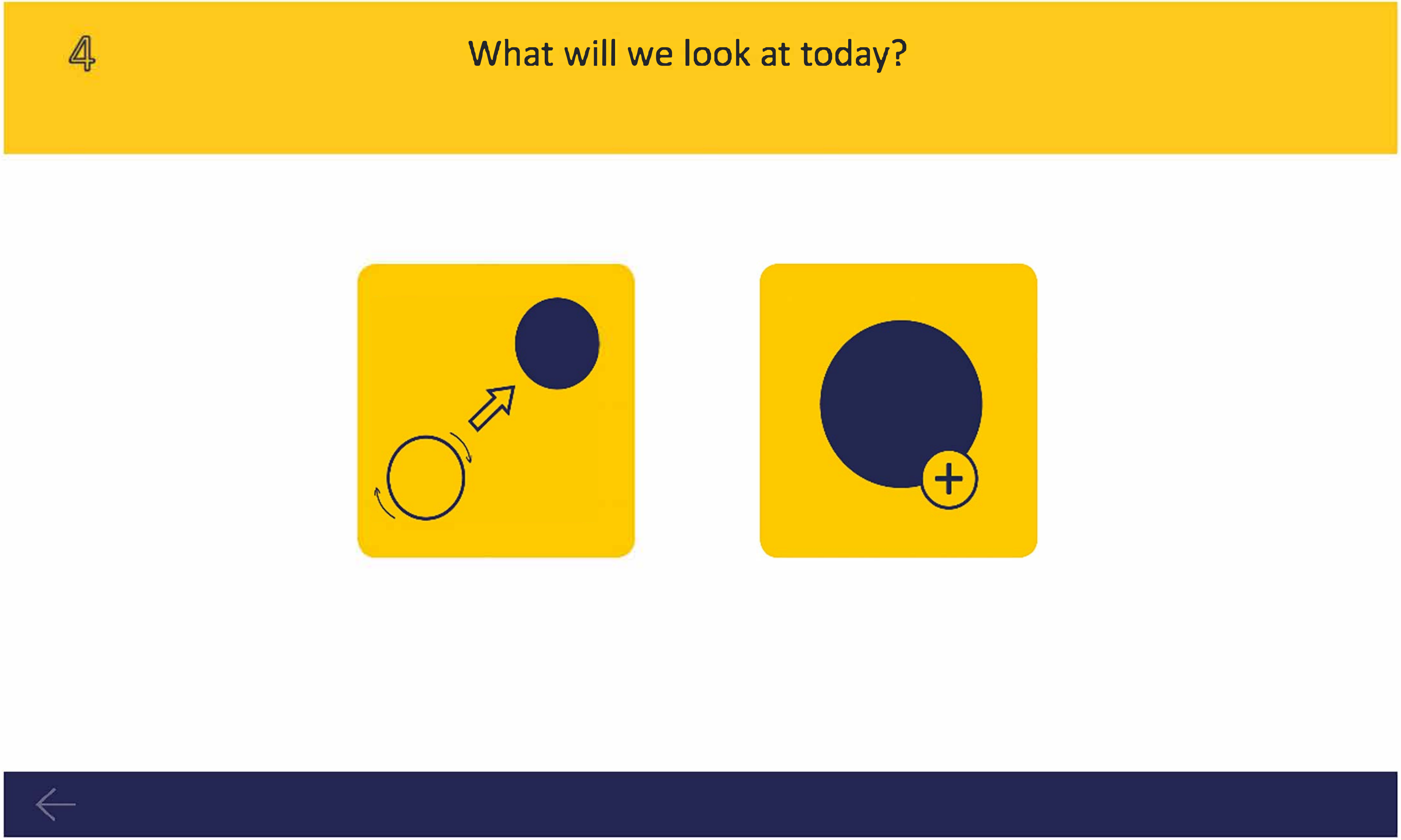

The aims screen displays boxes which the user taps to access an overview of the topics for the session.

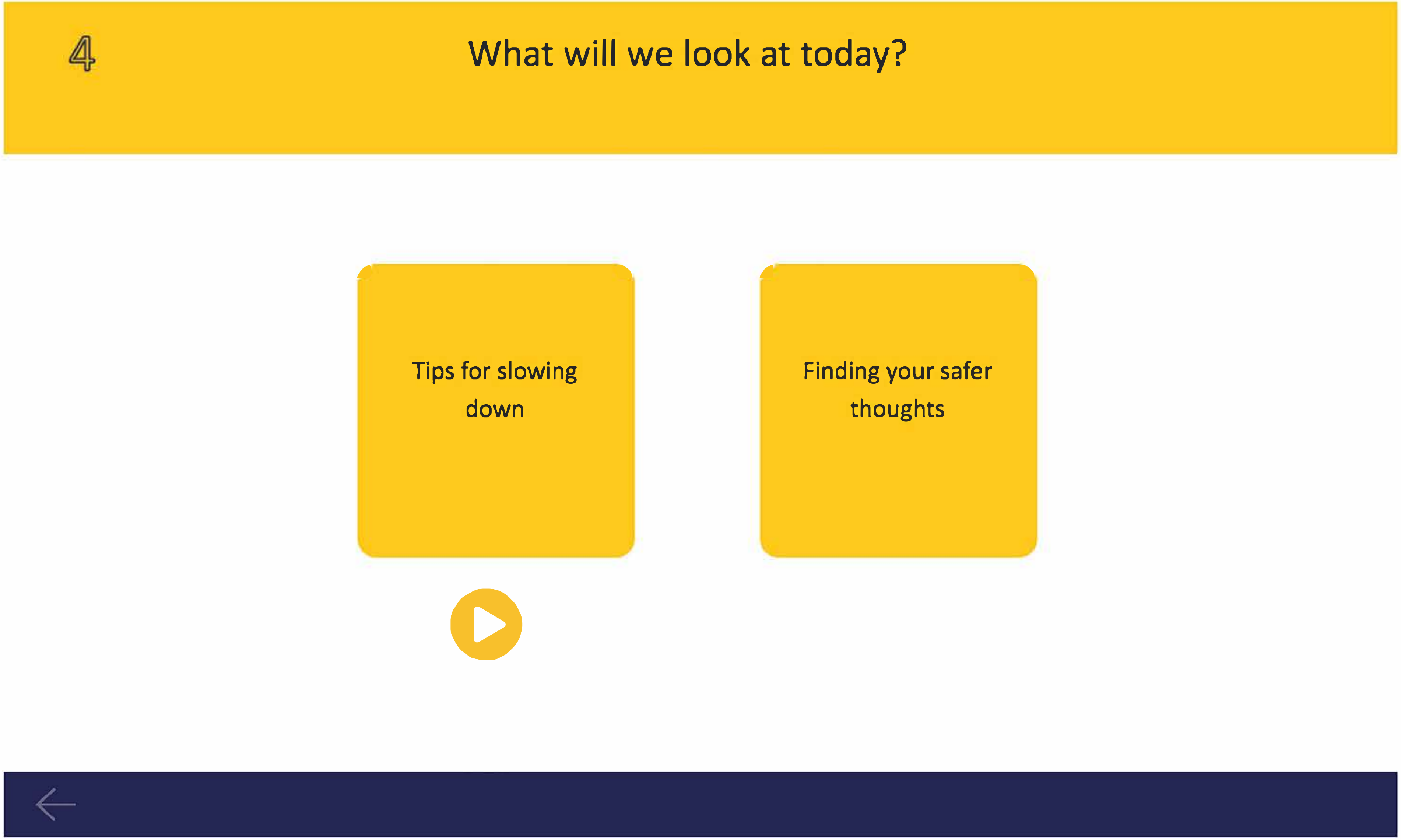

From the avatar screen, users access stories about other people's experiences of worries and how slowing down can help.

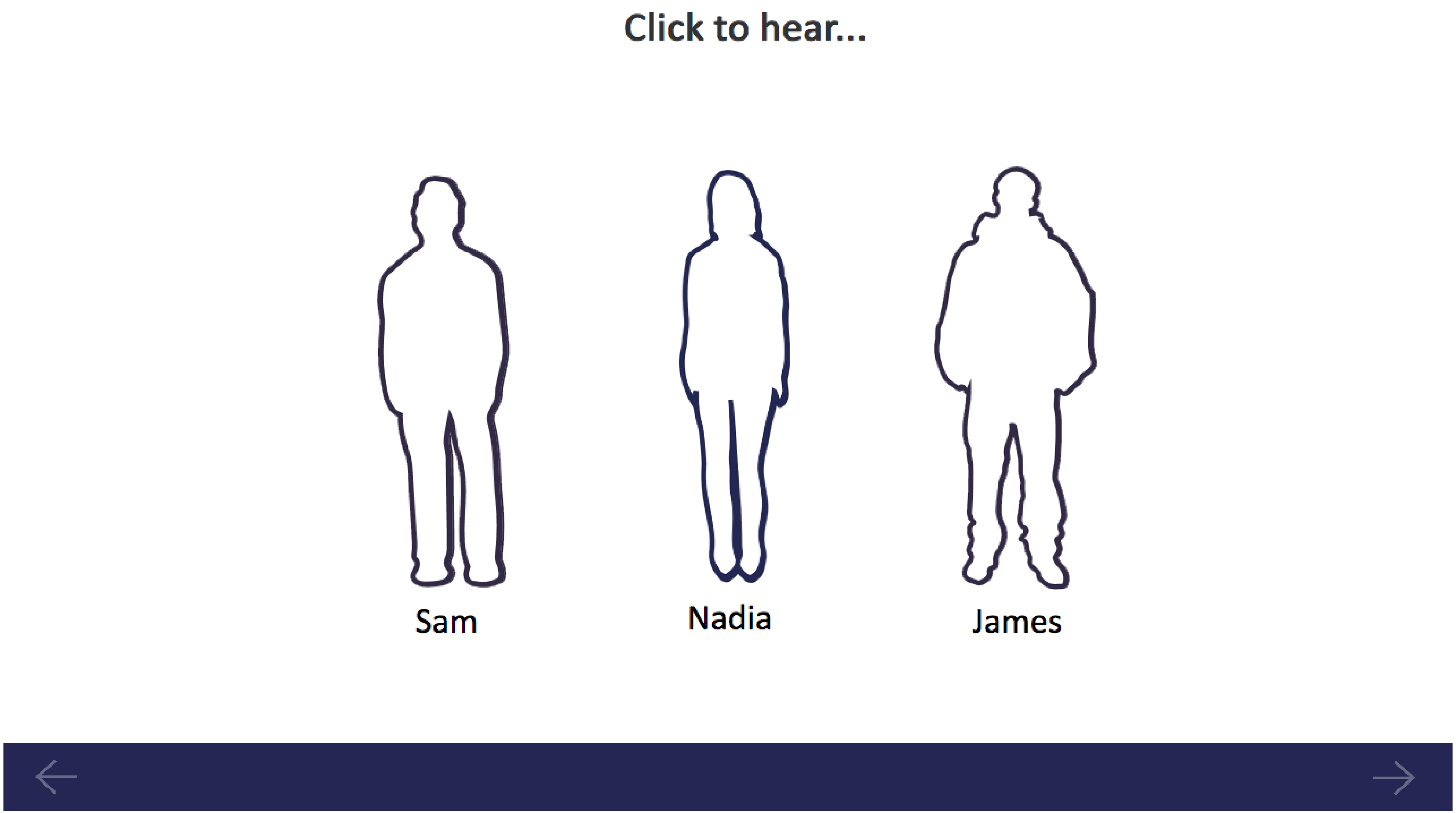

Example of a story from Nadia,
showing how difficult past experiences contribute to her feeling worried and unsafe in the present.

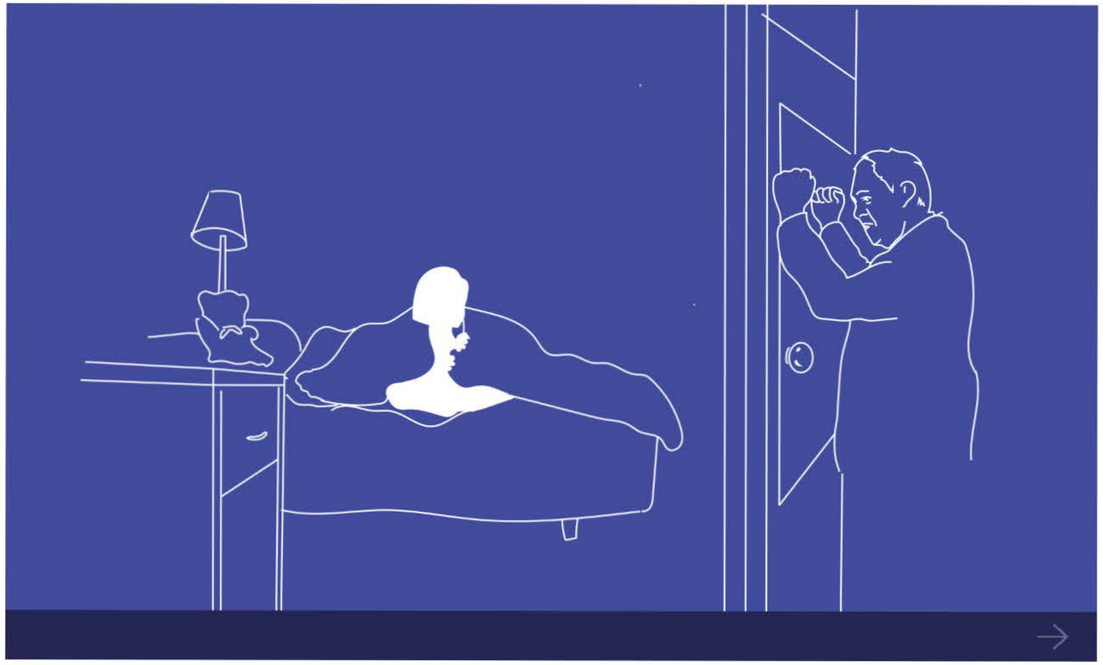

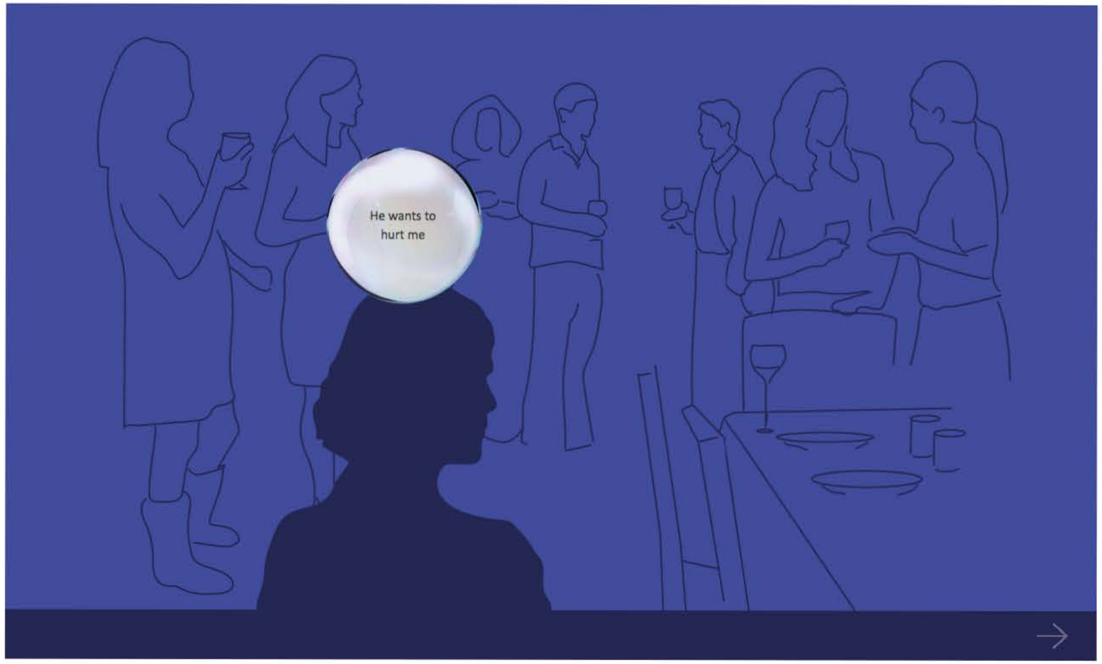

An example of psychoeducation information. A brief audiovisual animation highlights common worries, and how using tips to slow down thinking can shrink distressing thoughts and lead to safer, alternative ideas.

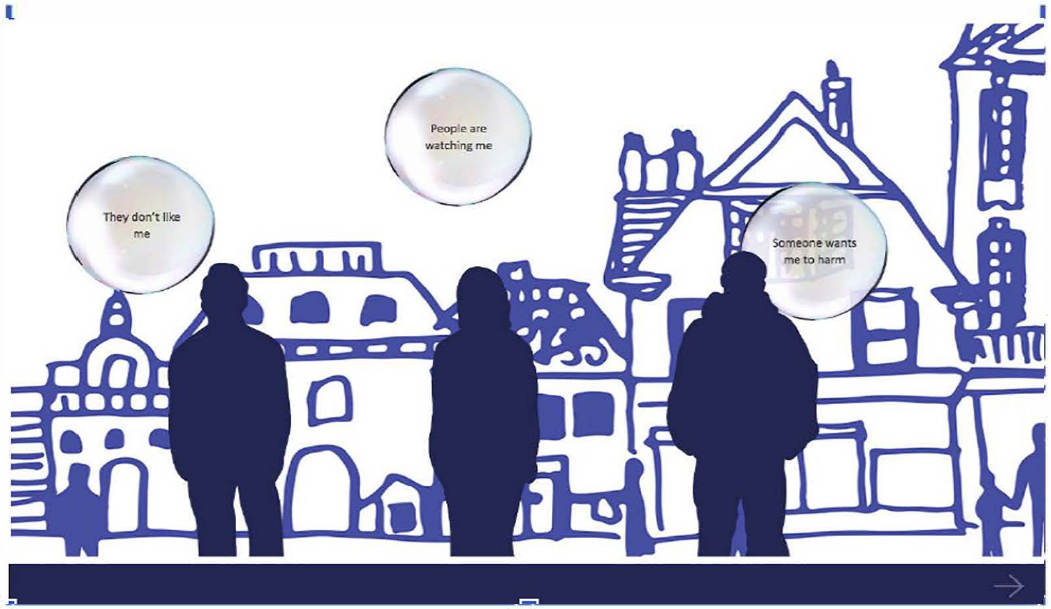

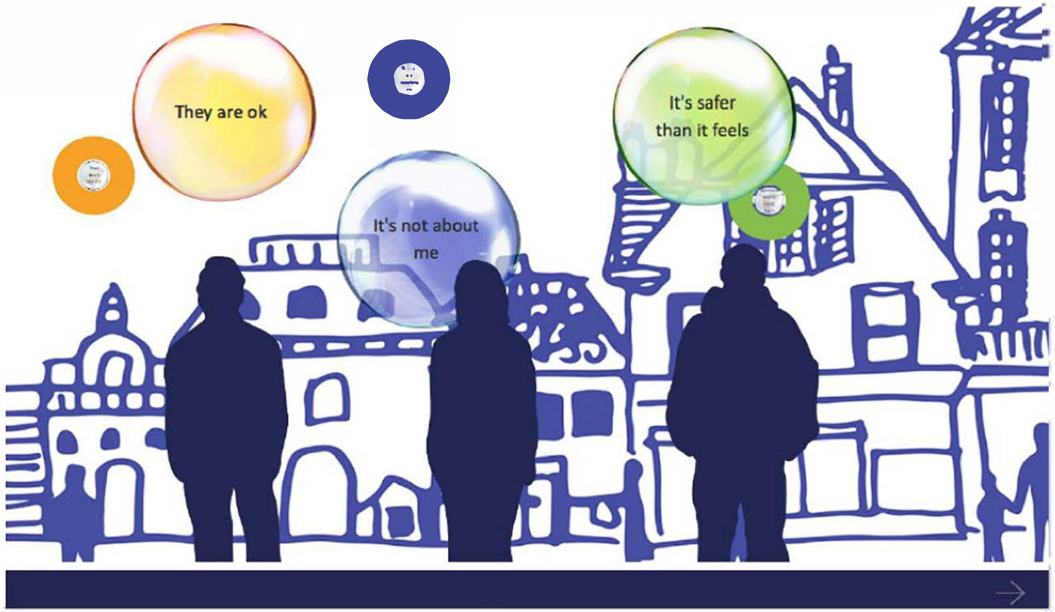

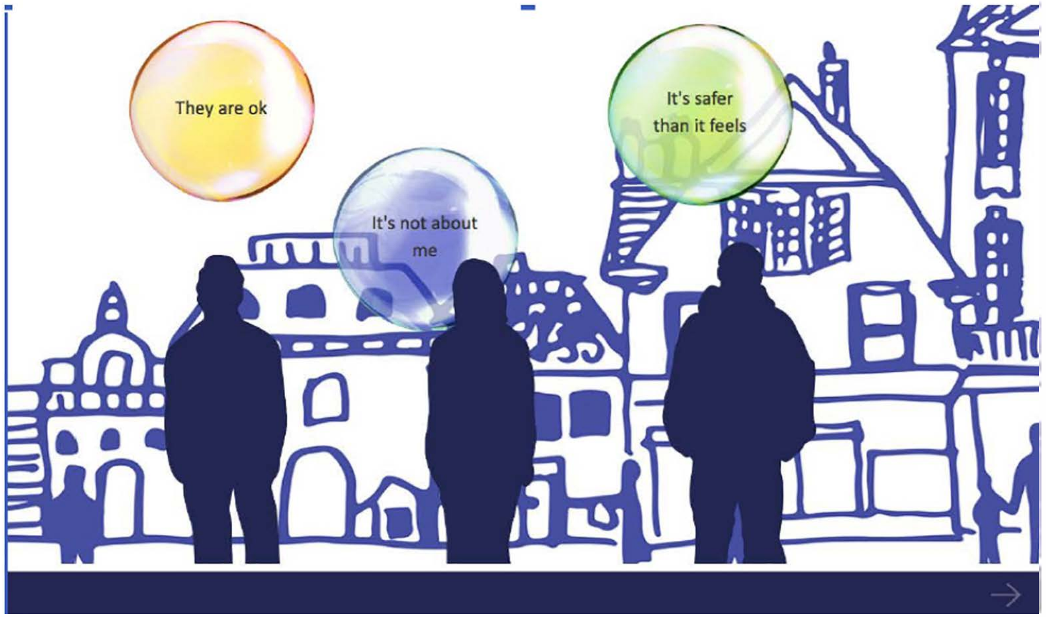

Example of a task to support users in applying psychoeducation to manage their worries.
UăŜŶă first select a worry from their formulation so they can practice slowing down ŭKŜŴ thinking.

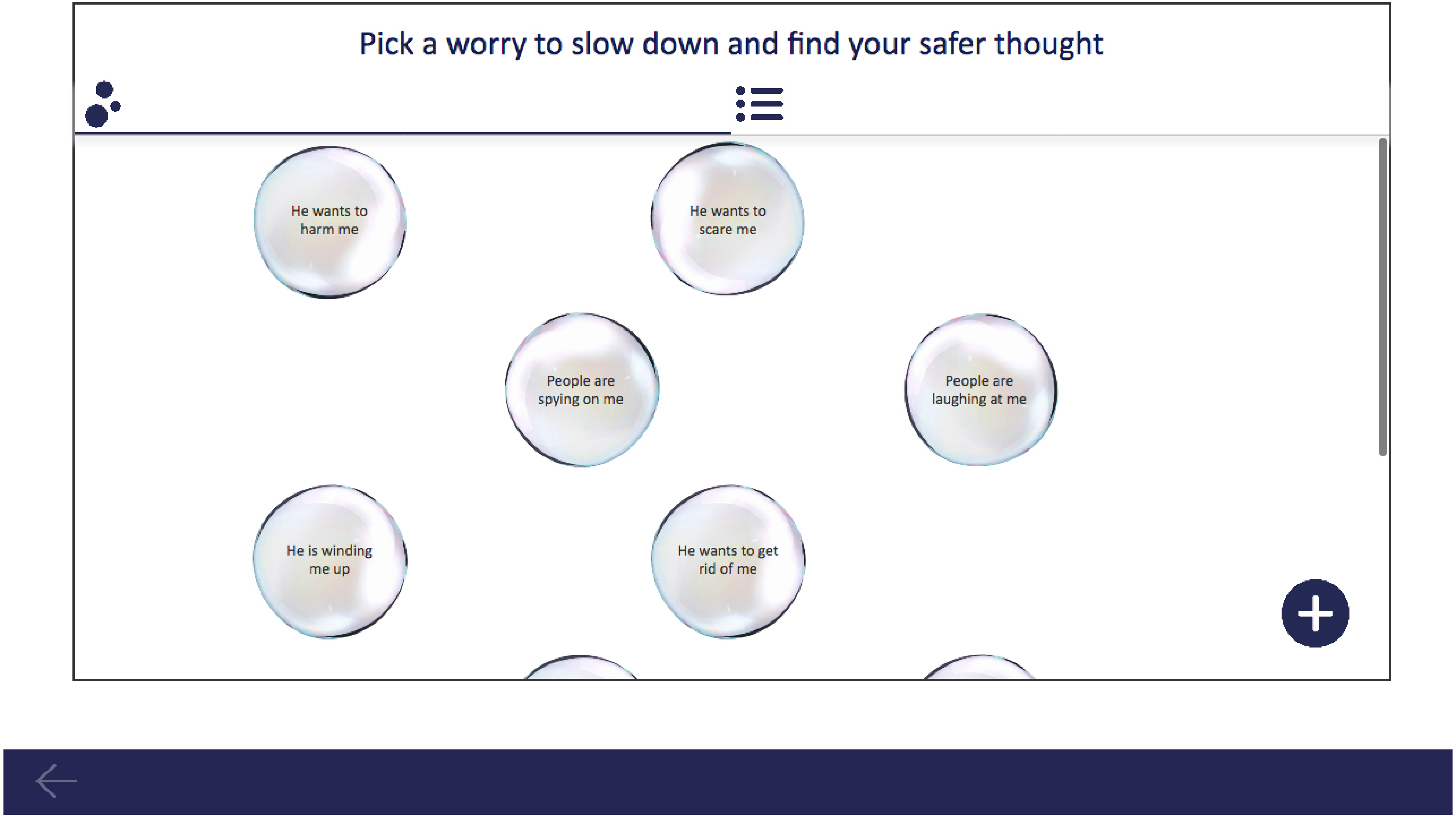

Example of a task to support users in applying psychoeducation to manage their worries.
[illegible] practice slowing down [illegible] thinking in relation to a selected worry [illegible] [illegible] they have learnt about during the session.

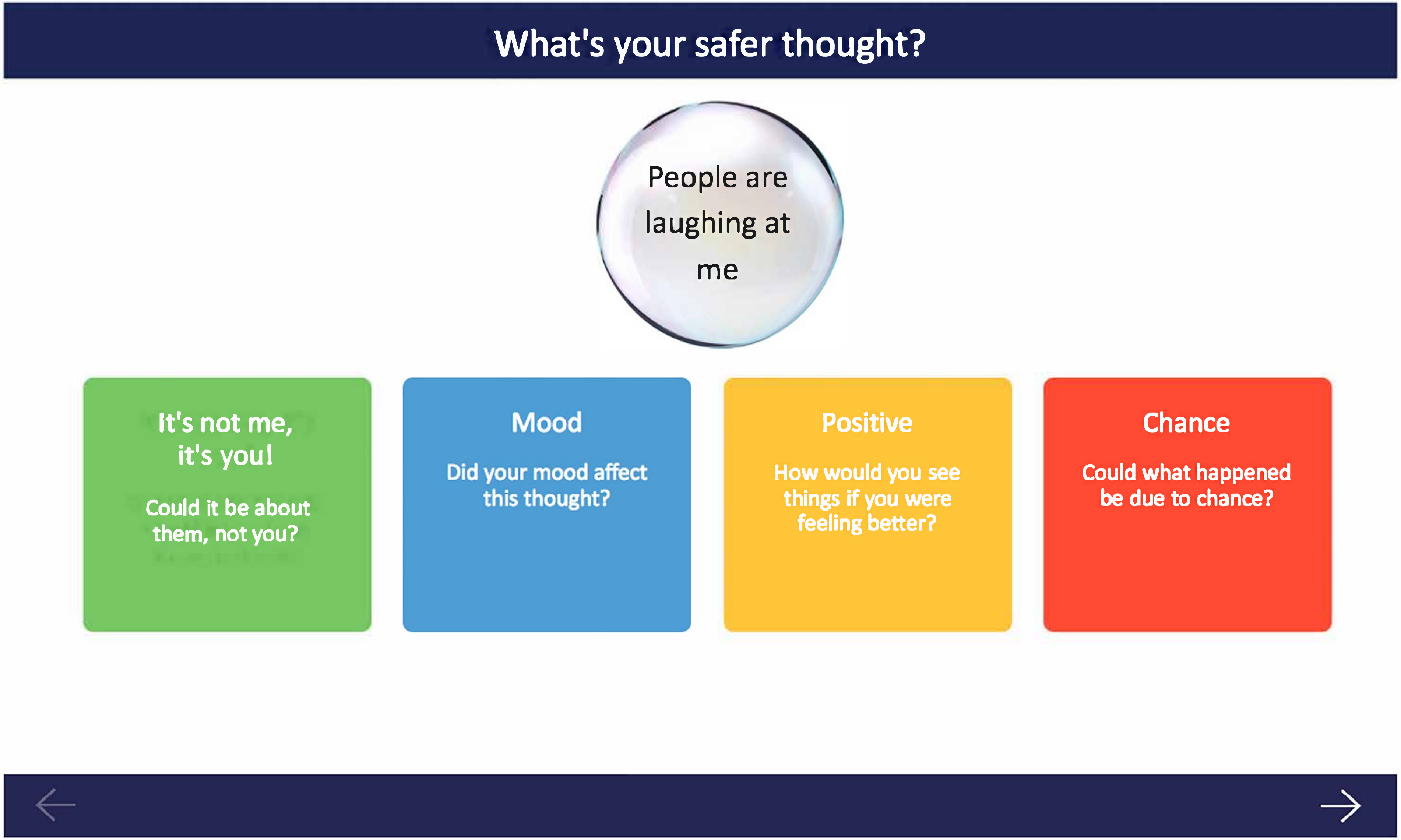

Mobile app content is unlocked towards the end of each session, based on the session content.
An app guide provides an overview of the new functionality.

4

Finding your safer thoughts

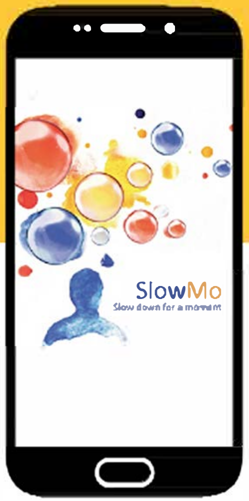

SlowMo

App guide

After reviewing the new mobile app content, the webapp then prompts the user and their therapist to practice using the new content during the session, outside of the consulting room where possible.

Let's have a go

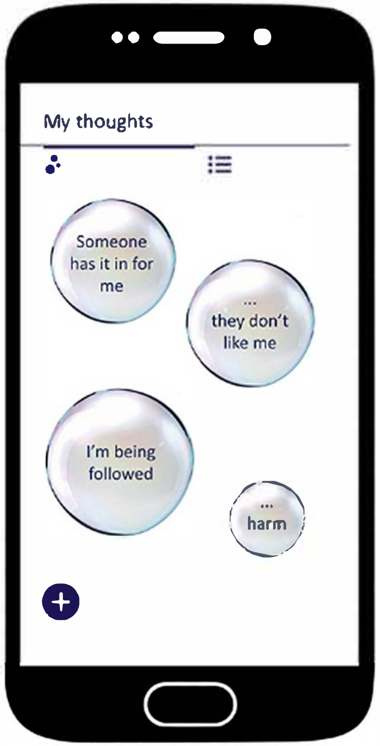

Towards the end of each session, users audio or text record a message about what they have learnt. This can then be accessed from the burger menu of the mobile app.

4

What have you learnt today?

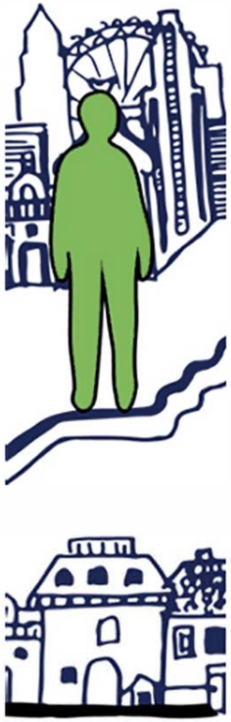

record a message

write a note

Towards the end of each session, users audio or text record a message about what they want to do before next session in relation to their therapy goal. This can then be accessed from the burger menu of the mobile app.

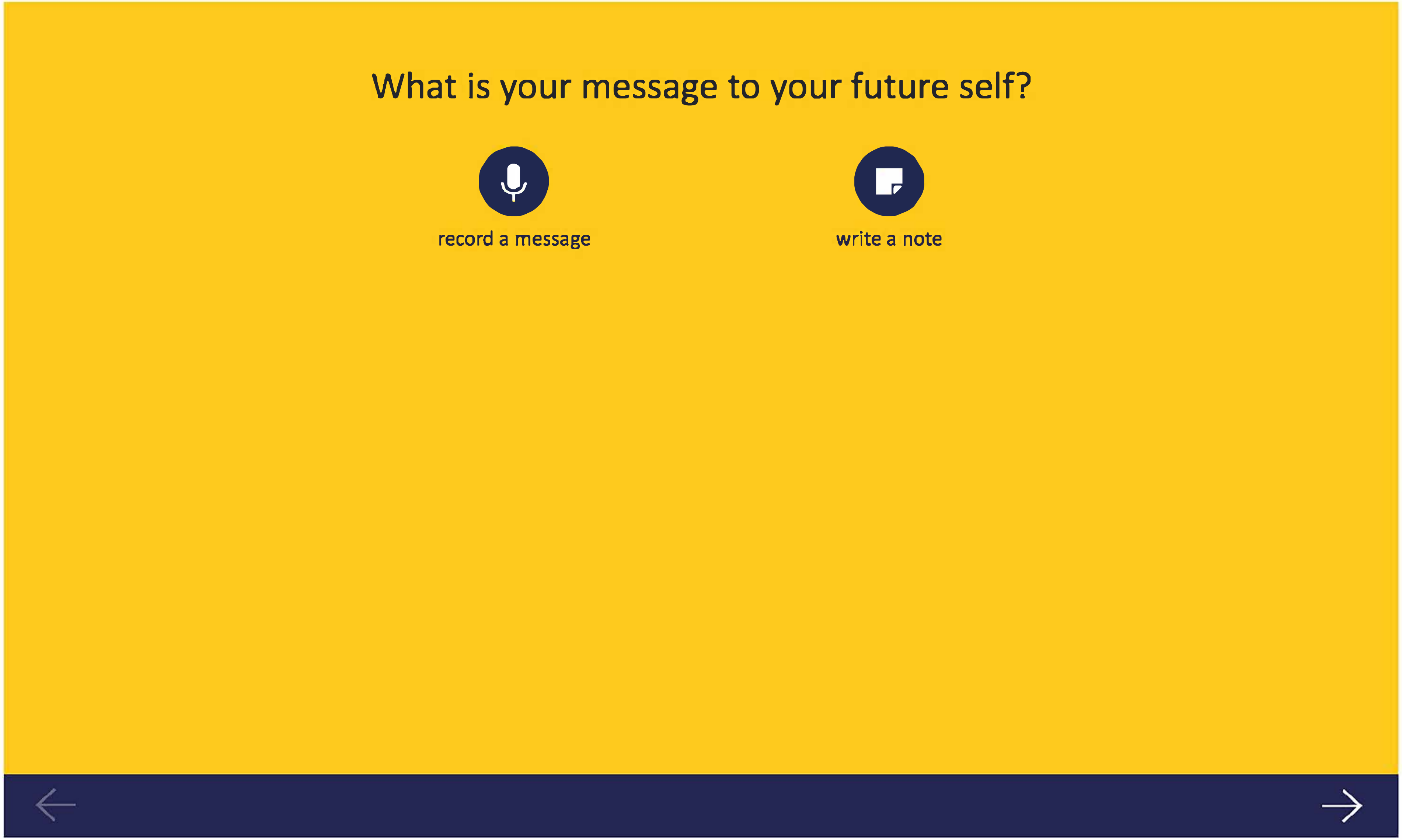

During the final session, graphs collate the weekly ratings of distress, conviction and thinking habits so users can review their progress.

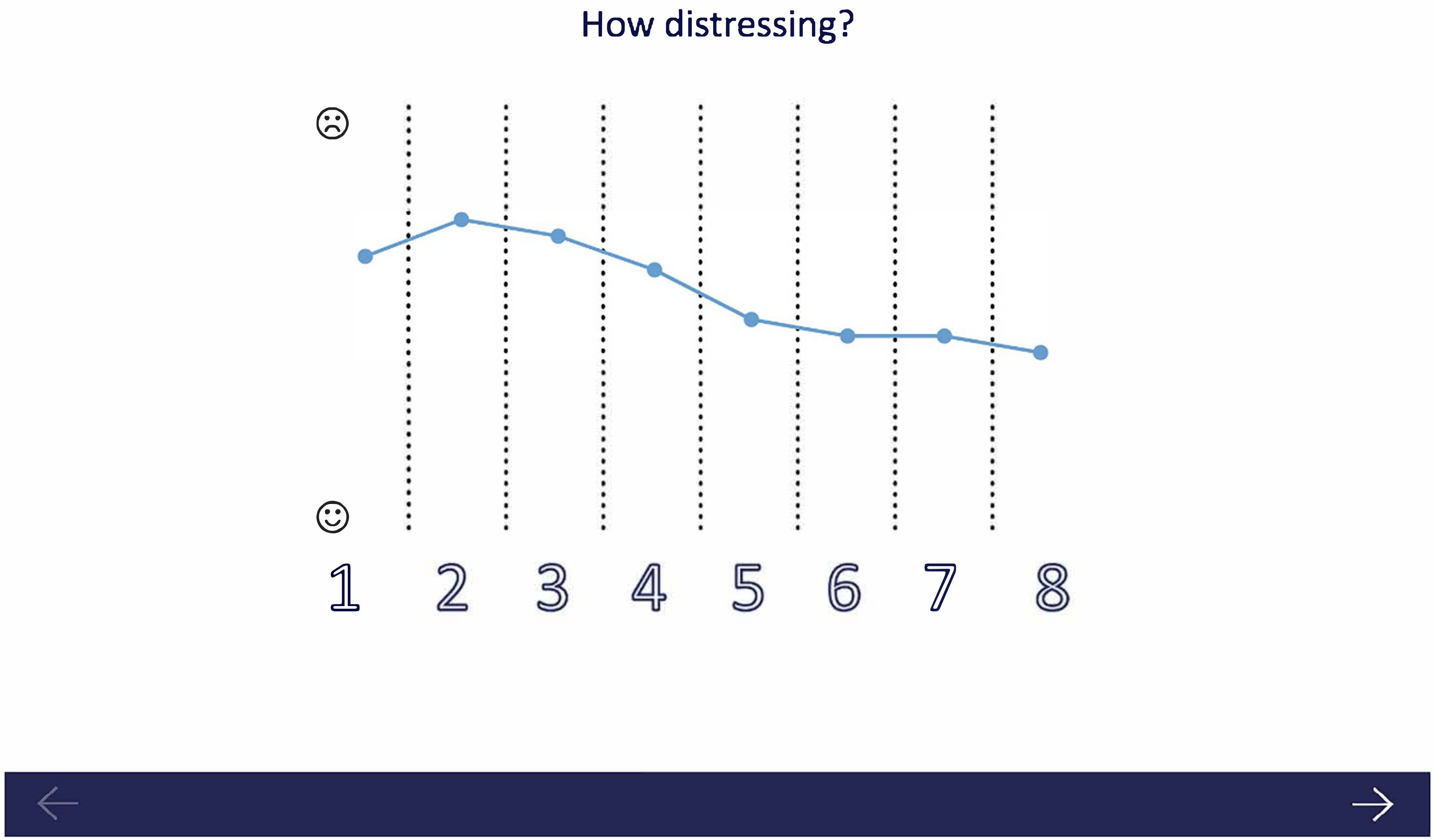

During the final session, users can customise the slowing down tips displayed on the mobile app according to their personal preferences.

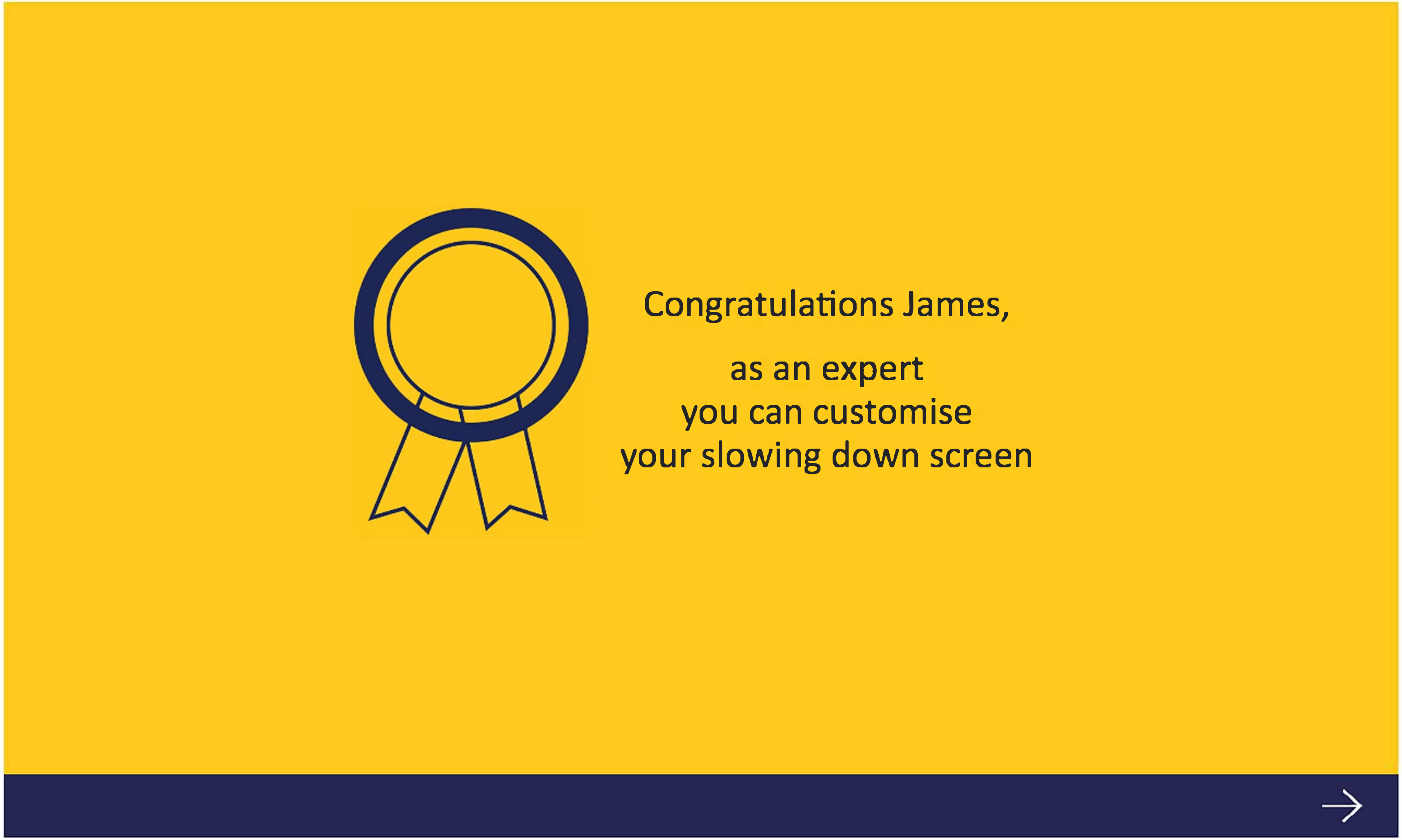

During the final session, users can customise the slowing down tips displayed on the mobile app according to their personal preferences.

My slowing down tips

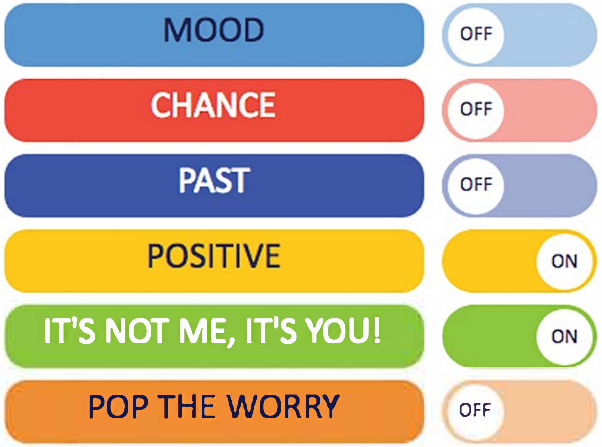

The journey home screen displays when the user has finished a session, with the completed therapy journey shown here.

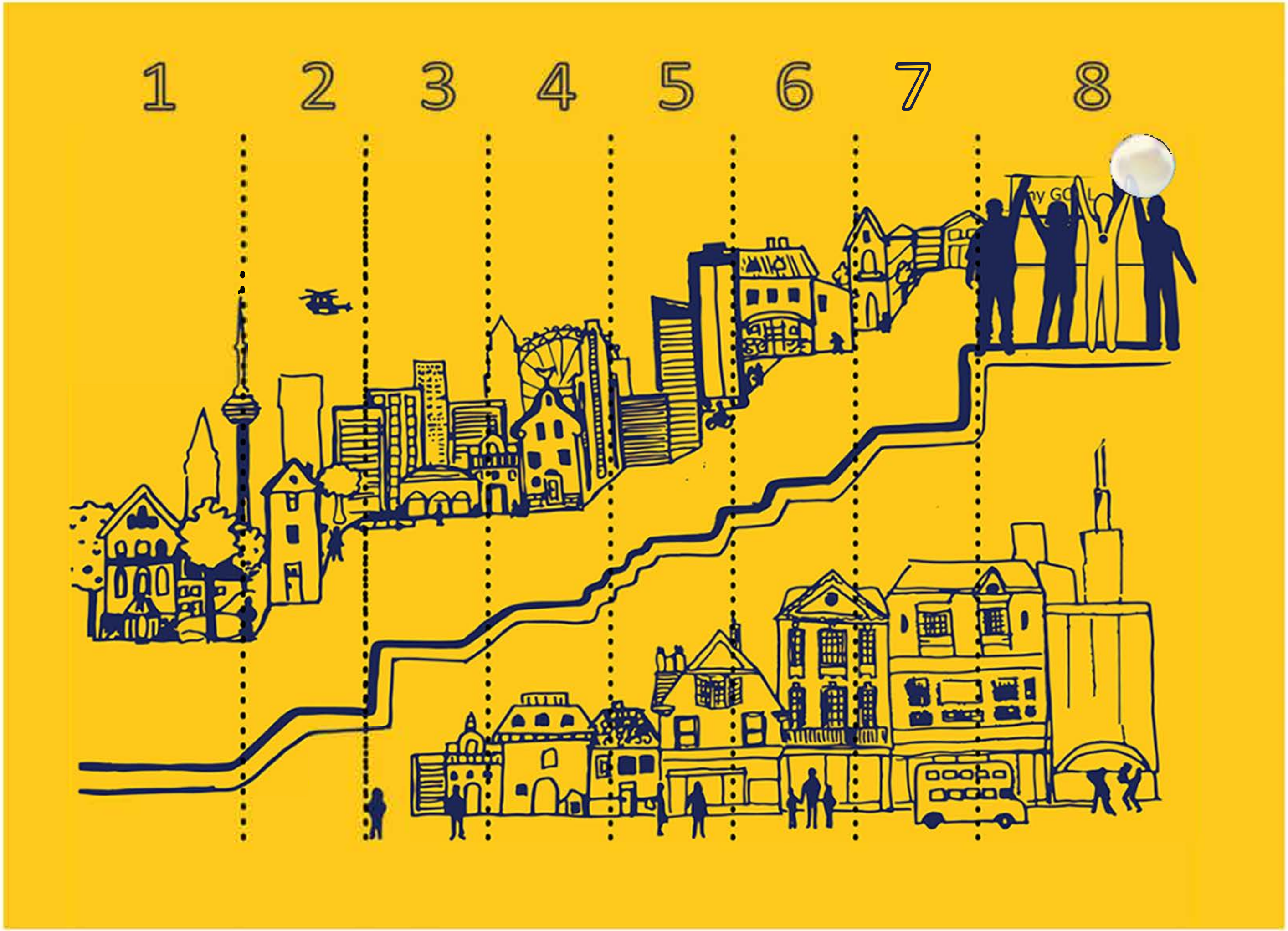

Supplement: Multimedia Appendix 4 [file mental_v5i4e11222_app4.pdf]
