# Supplementary material for: How Inclusive, User-Centered Design Research Can Improve Psychological Therapies for Psychosis: Development of SlowMo
Source: JMIR Ment Health. 2018 Dec 5;5(4):e11222. doi: 10.2196/11222 (PMC6300708; doi:10.2196/11222)

SlowMo native app. Home screen has two viewings modes, the user swipes between their worries and their safer thoughts

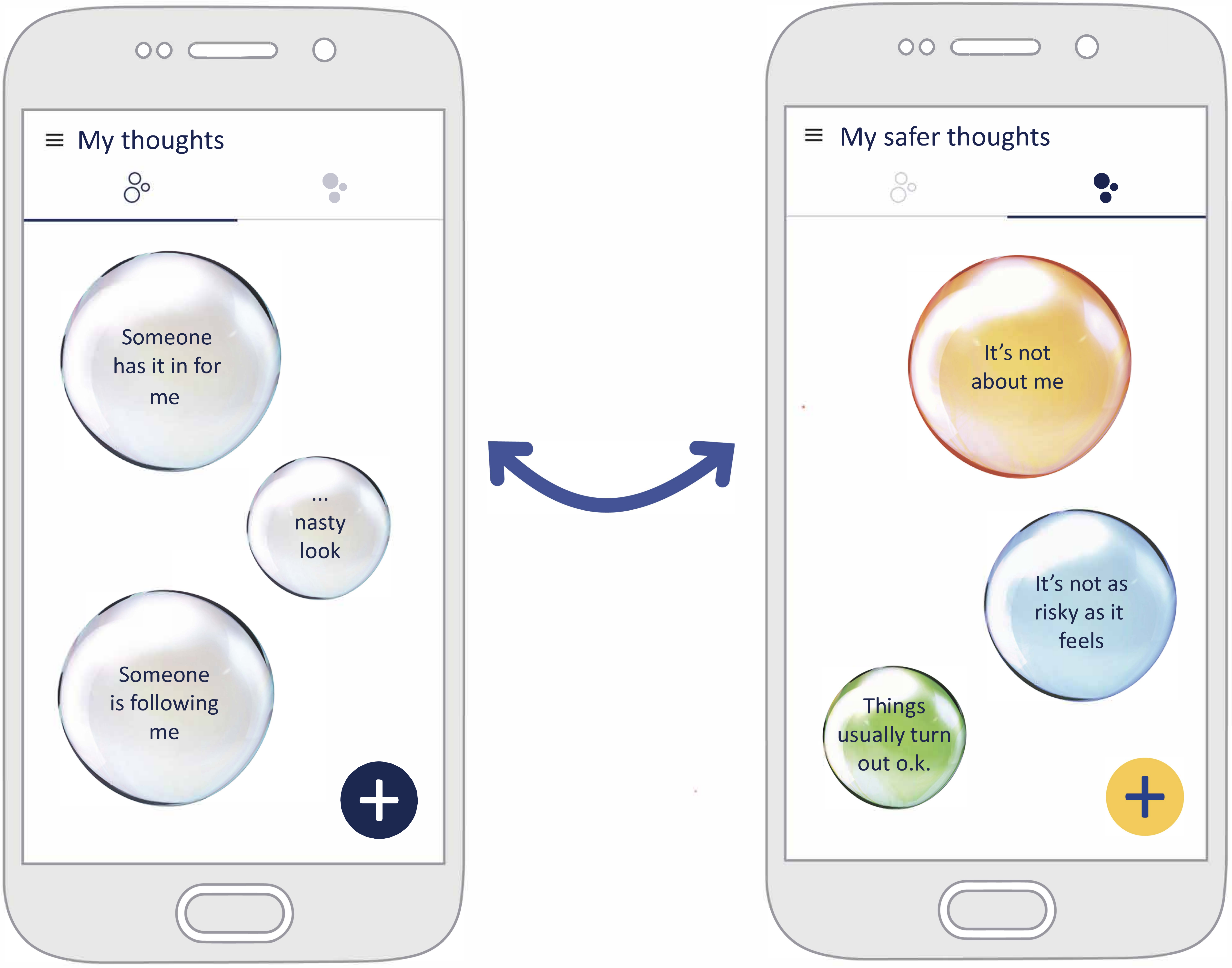

Following session one, the user taps on a bubble when the worry occurs, then sizes it to record the associated distress.

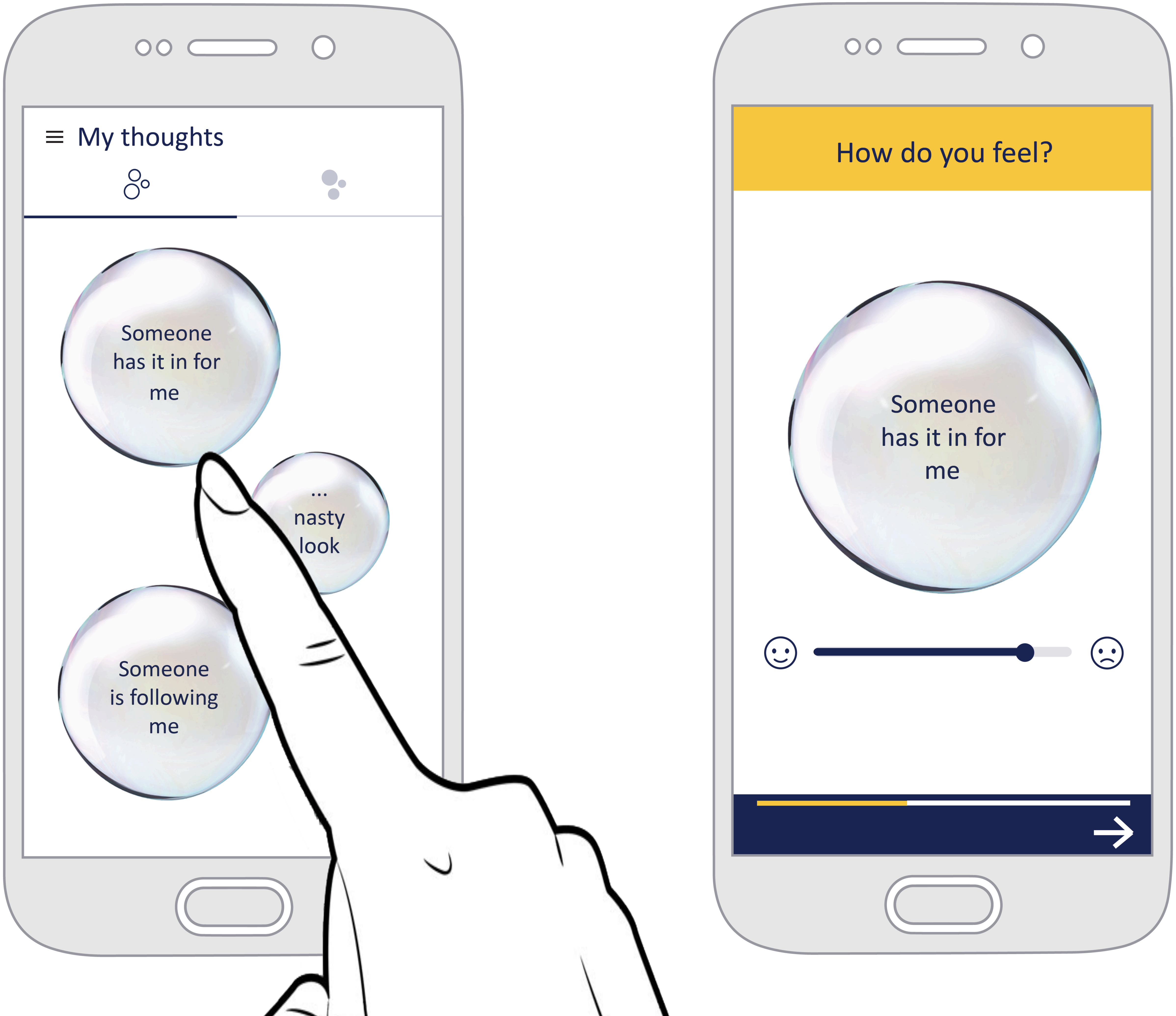

Following session two, users also record how fast or slow they are thinking in relation to the worry.

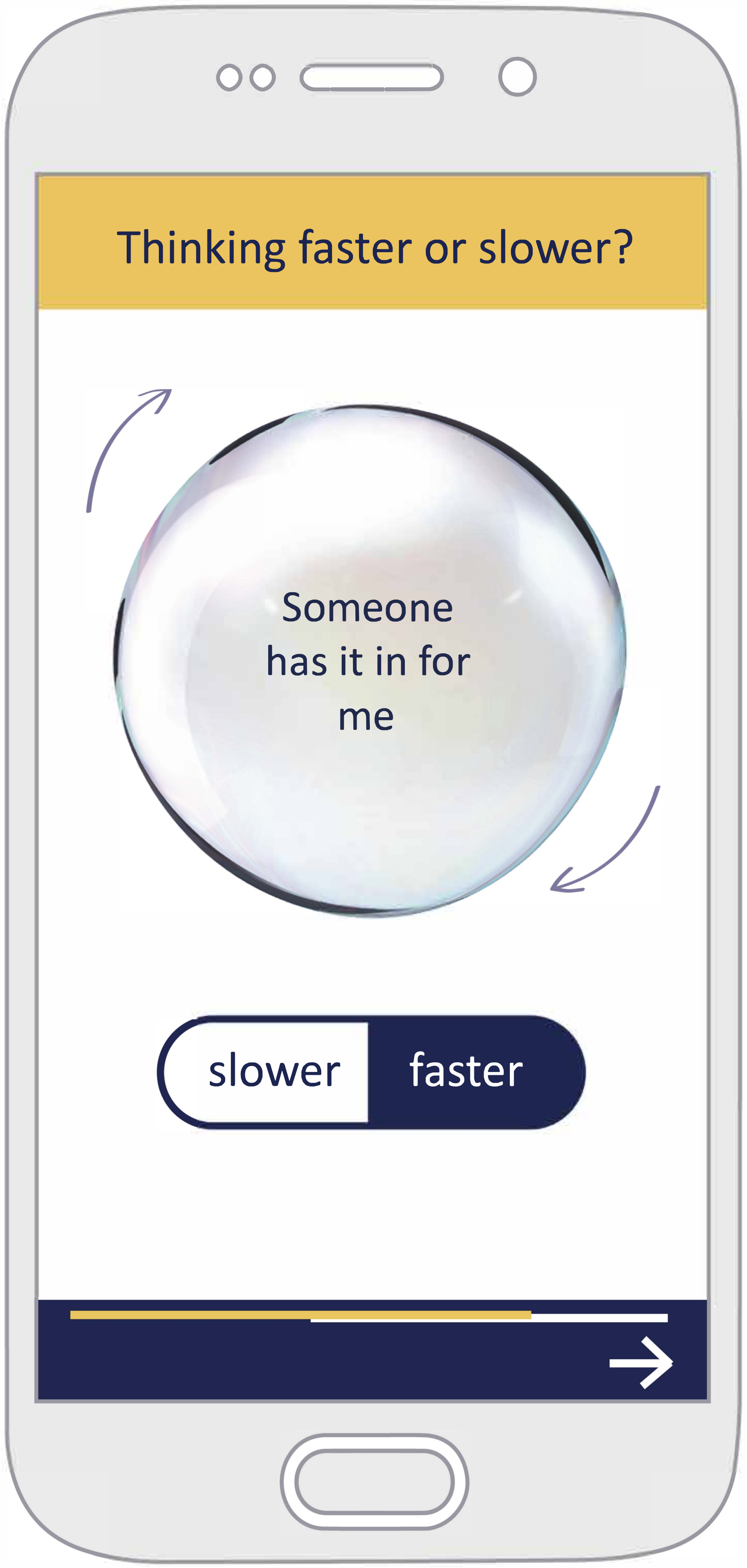

Following session three, users are encouraged to tap a spinning bubble to slow it down, to encourage reflective thinking.

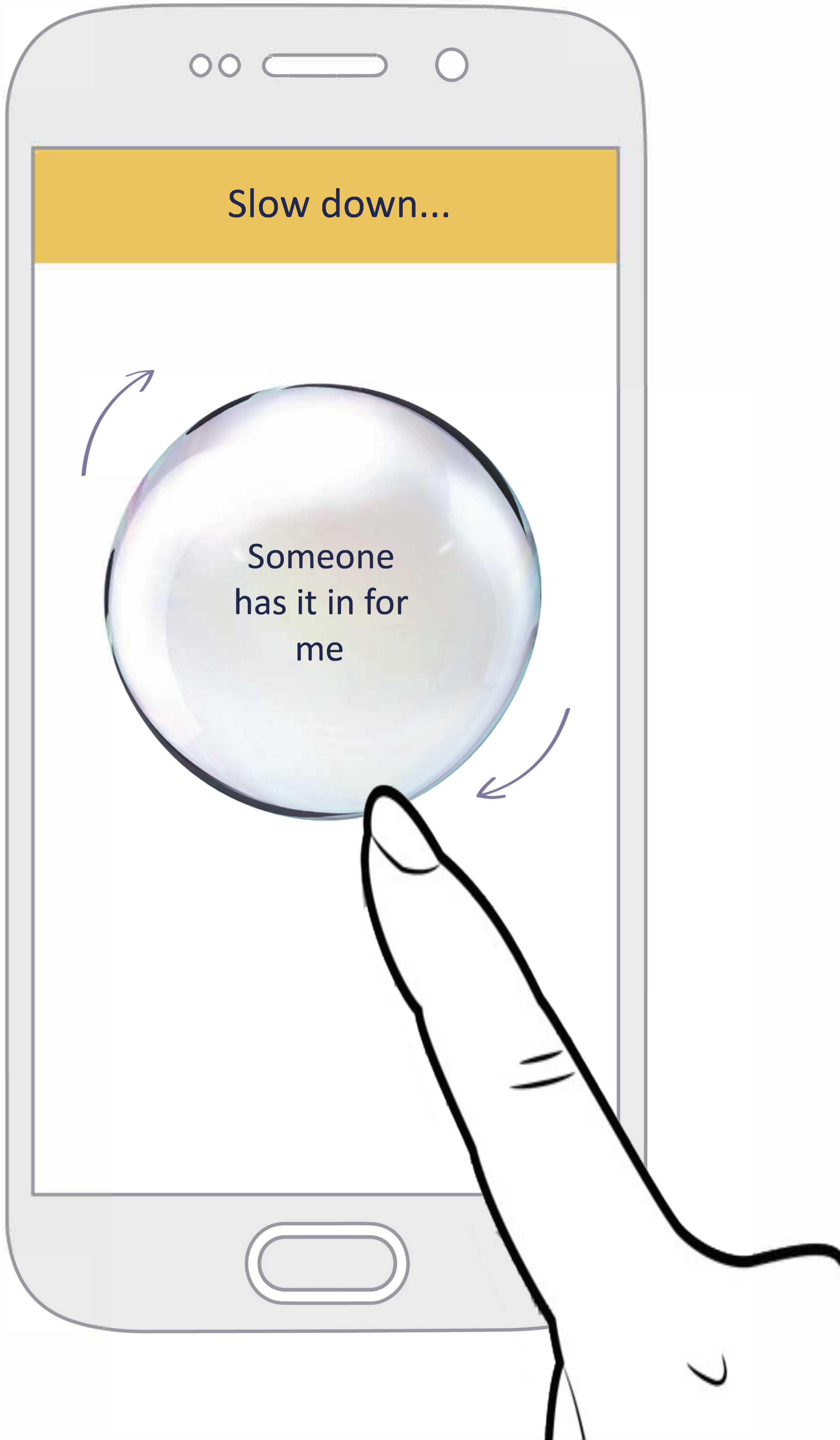

From session four onwards, tip cards are added, based on the learning in session, to support the user in slowing down, together with the option to audio or text record additional information.

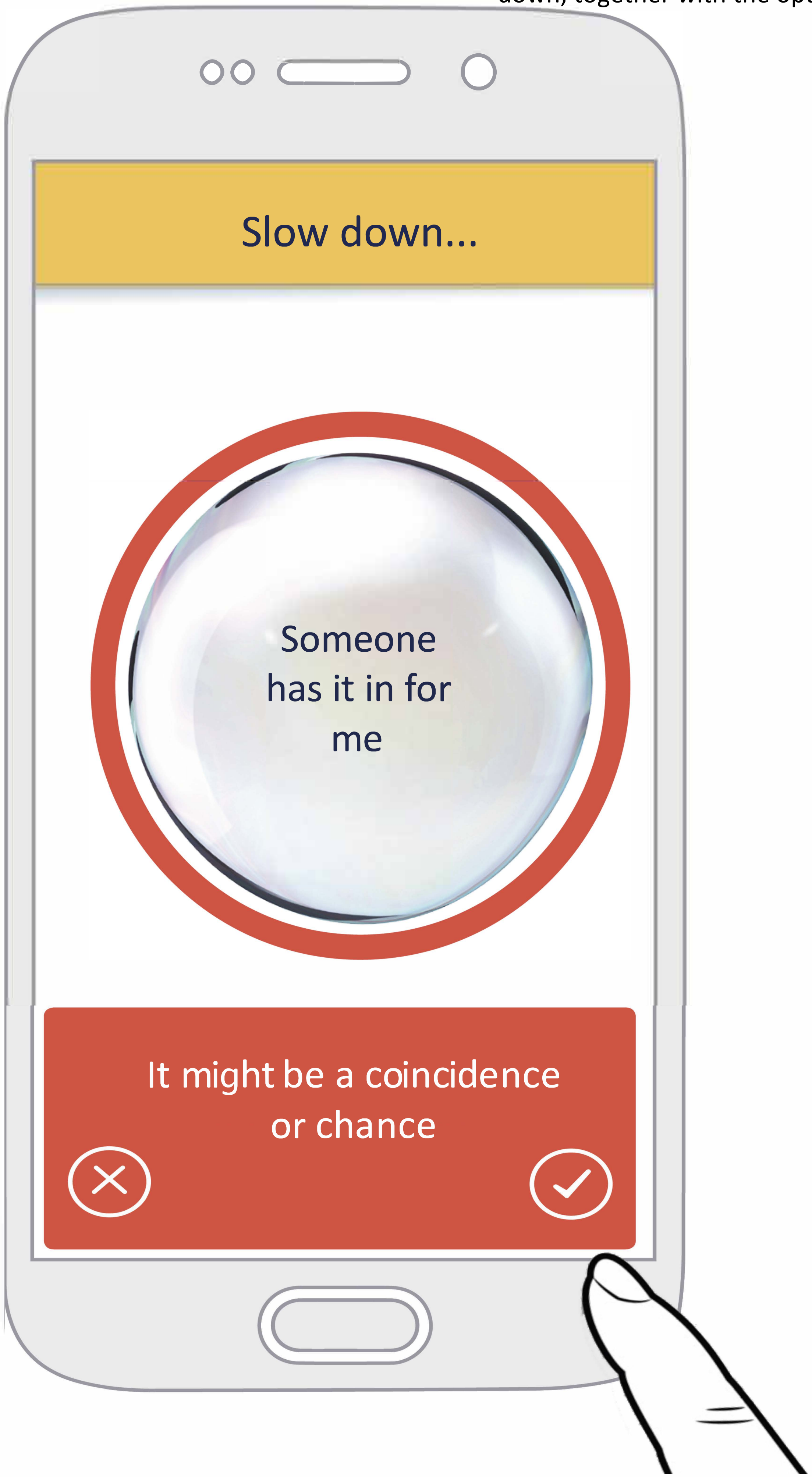

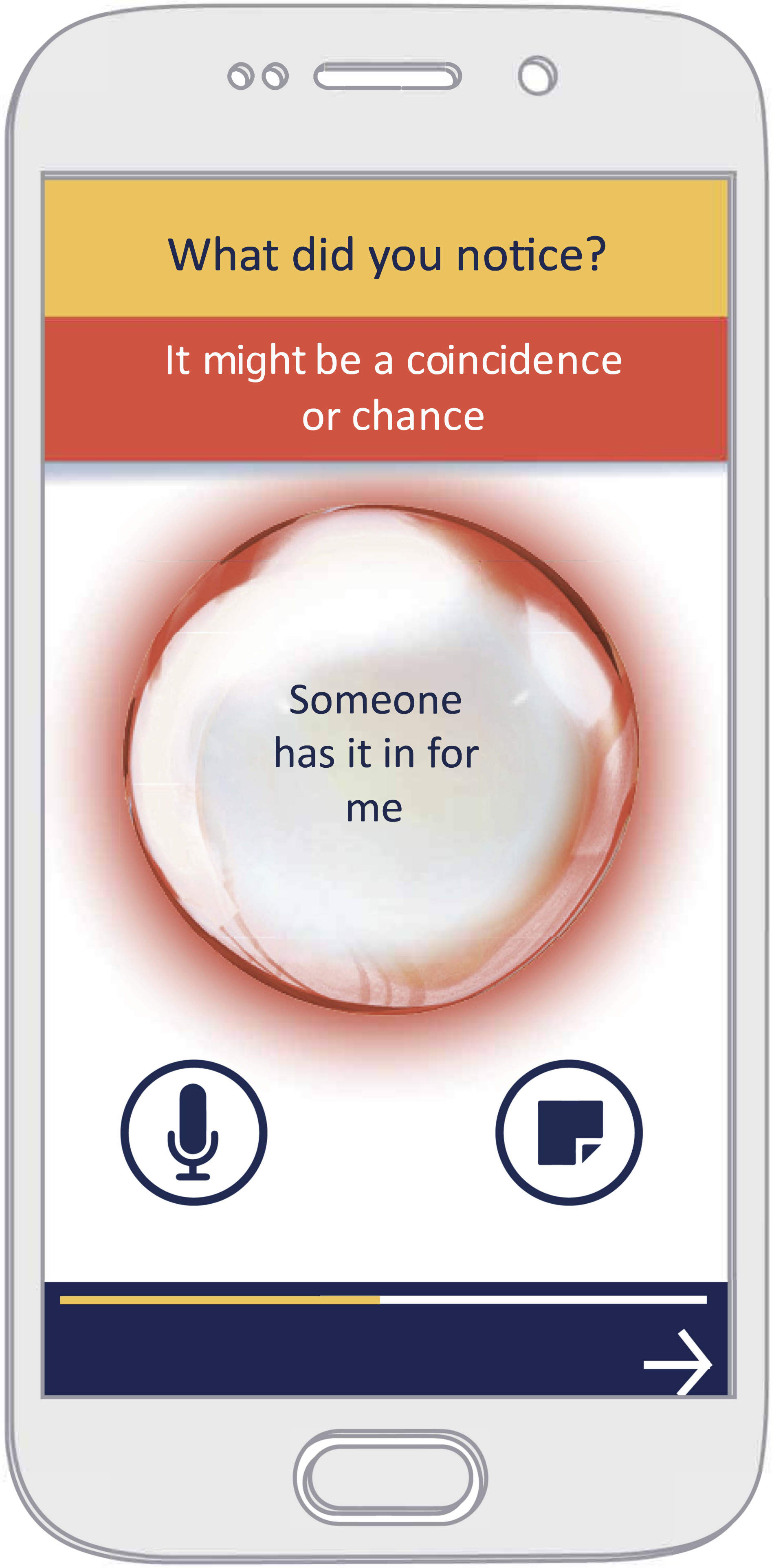

At the end of the slowing down process, the app supports the user to identify a safer thought and re-rate distress.

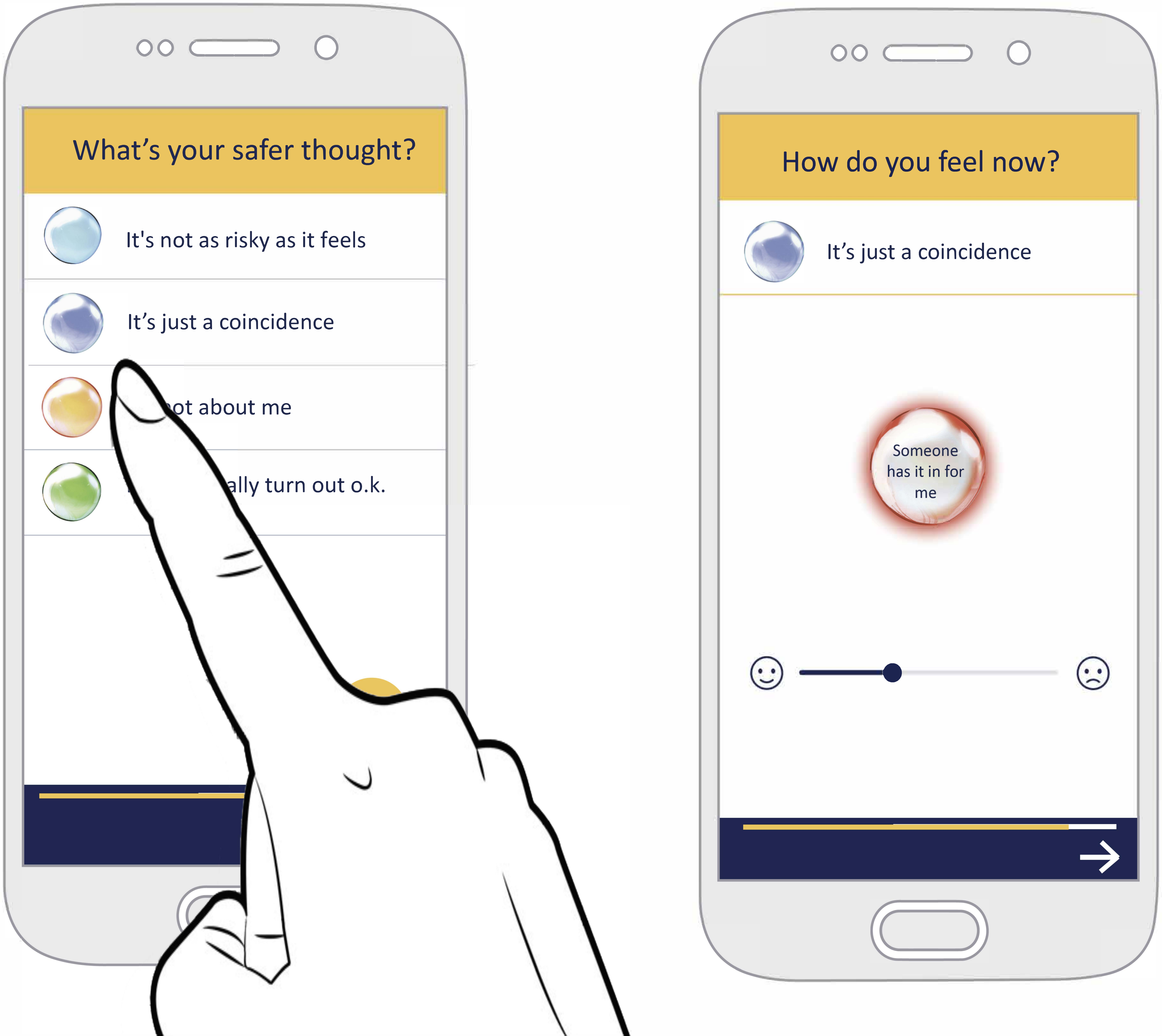

The home screen then updates, with the halo colour indicating the user has identified a helpful tip for the thought.

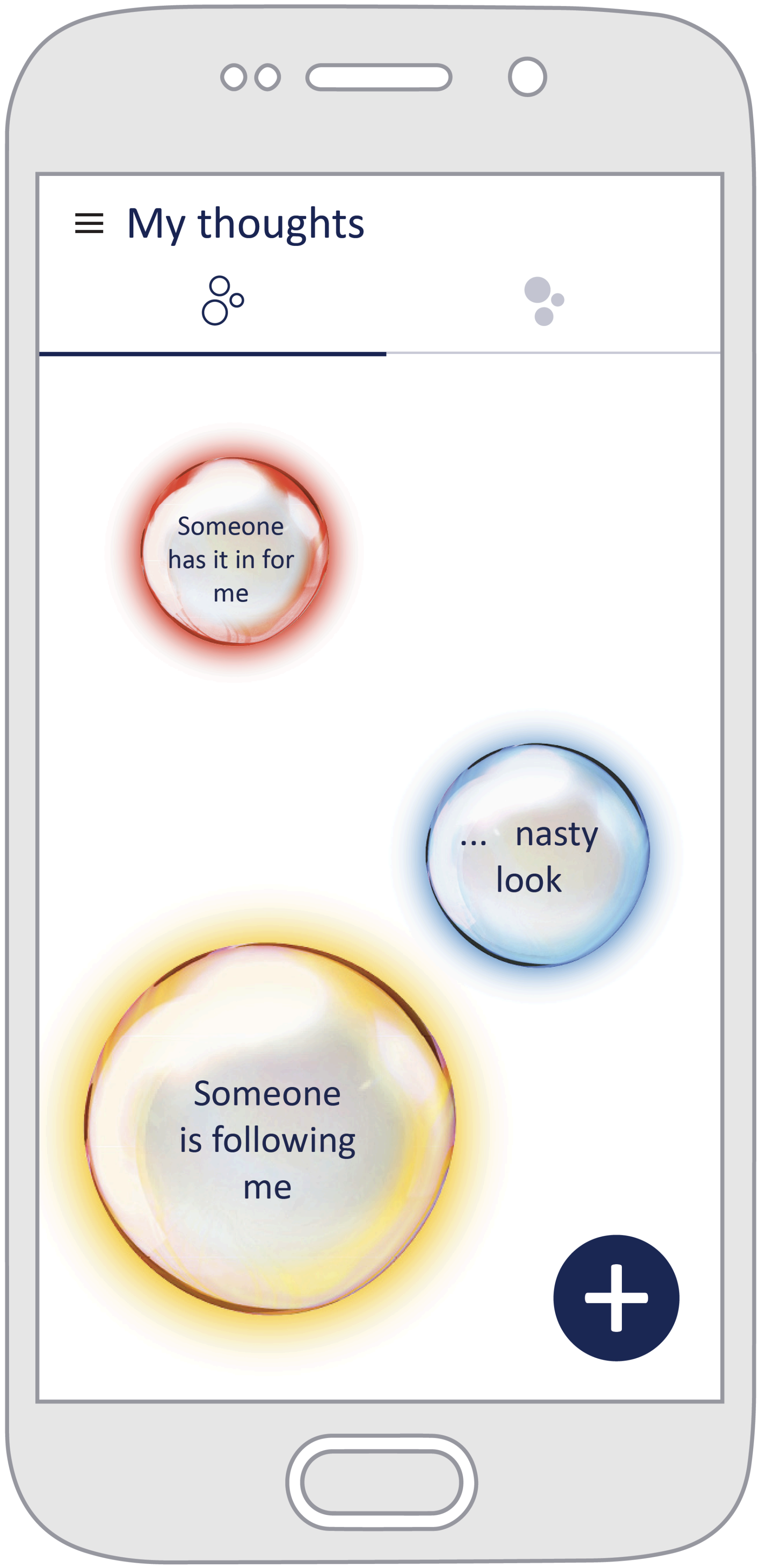

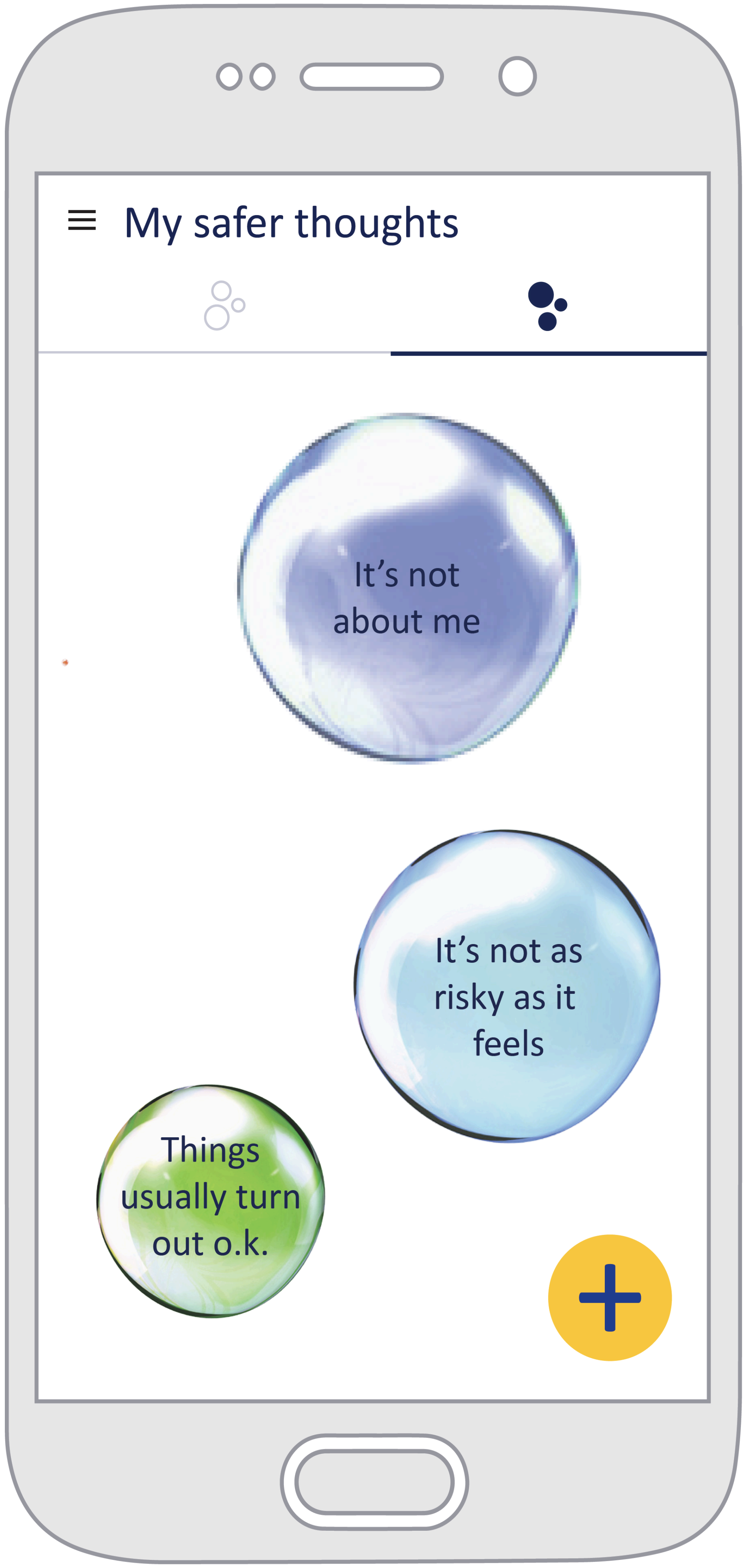

The thought profile screen records all previous instances of slowing down and the user's helpful tips for the worry.

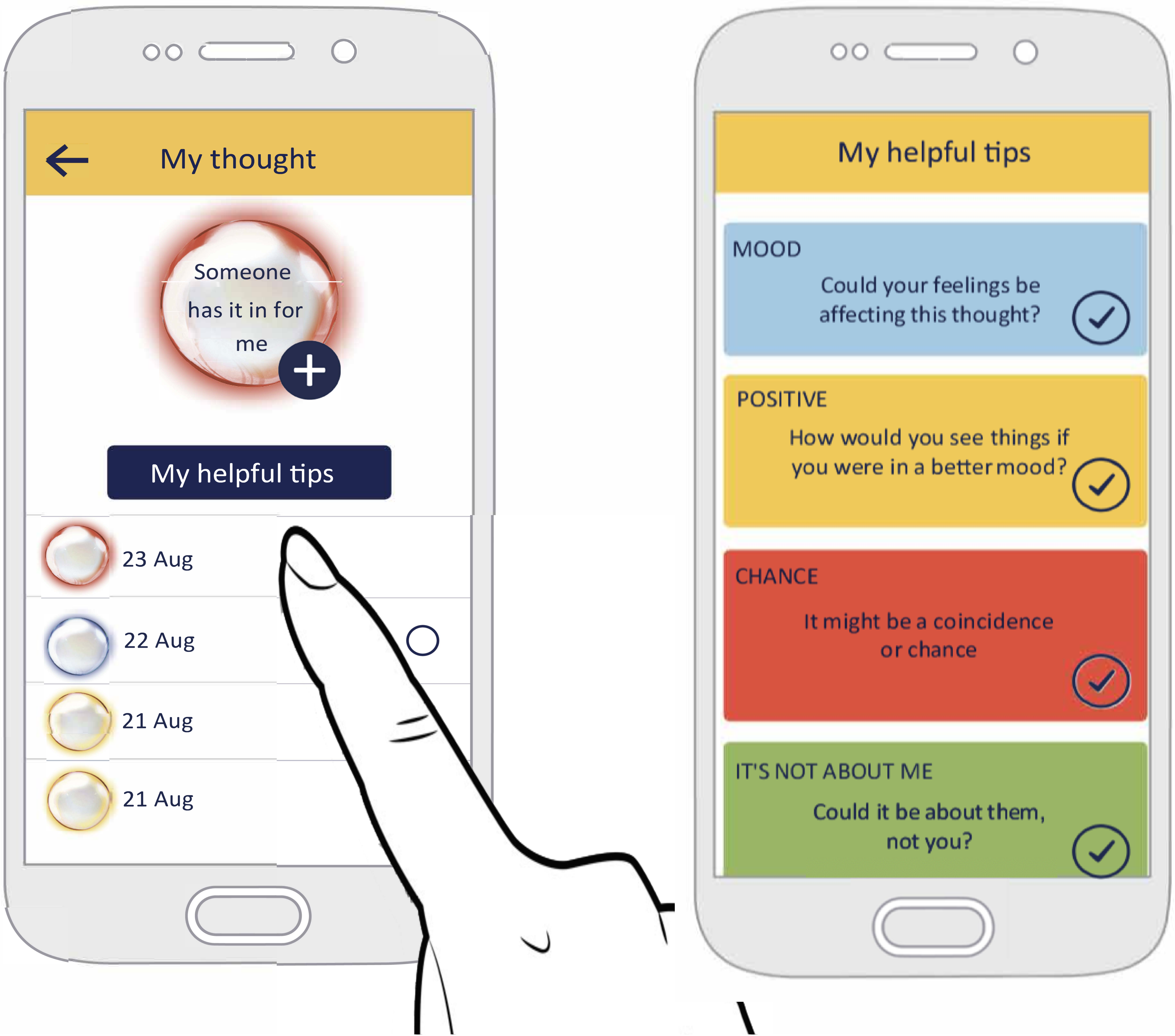

When the user taps on a previous instance of slowing down, a summary of what happened is displayed.

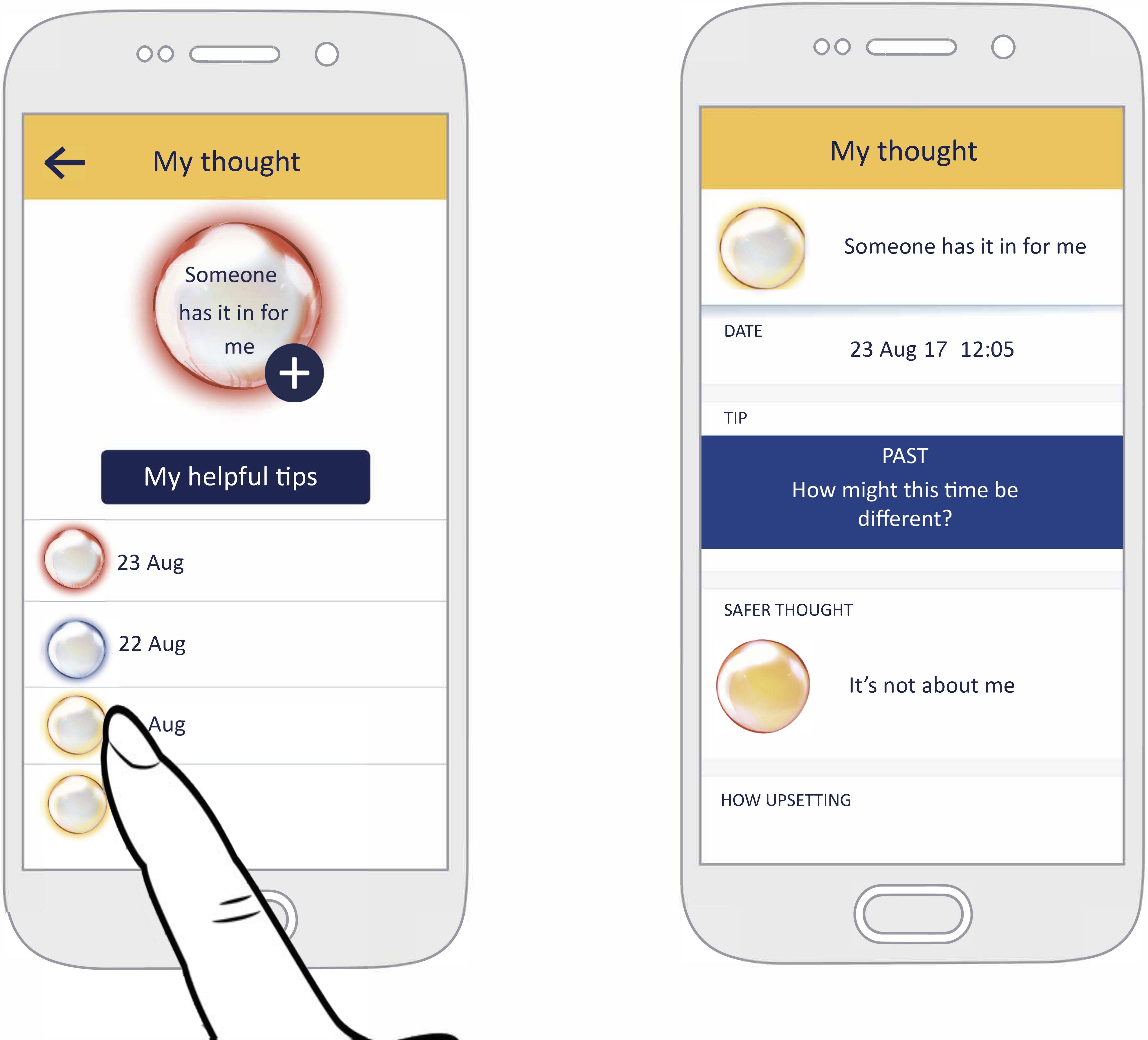

The burger menu opens to display settings, my journey session summaries, about SlowMo and my safety plan.

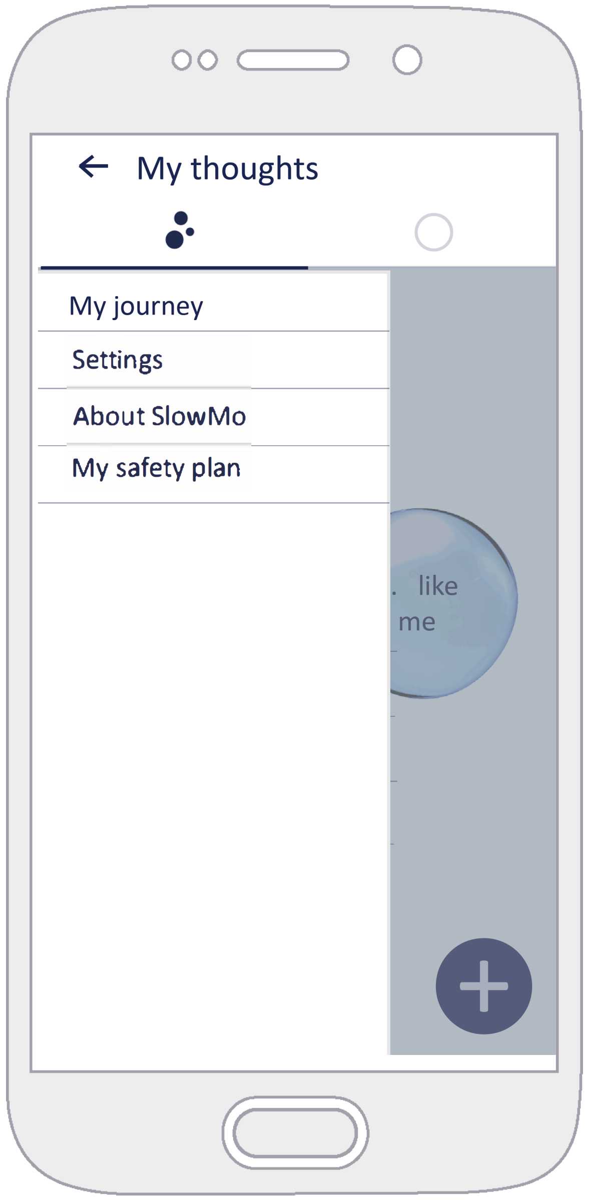

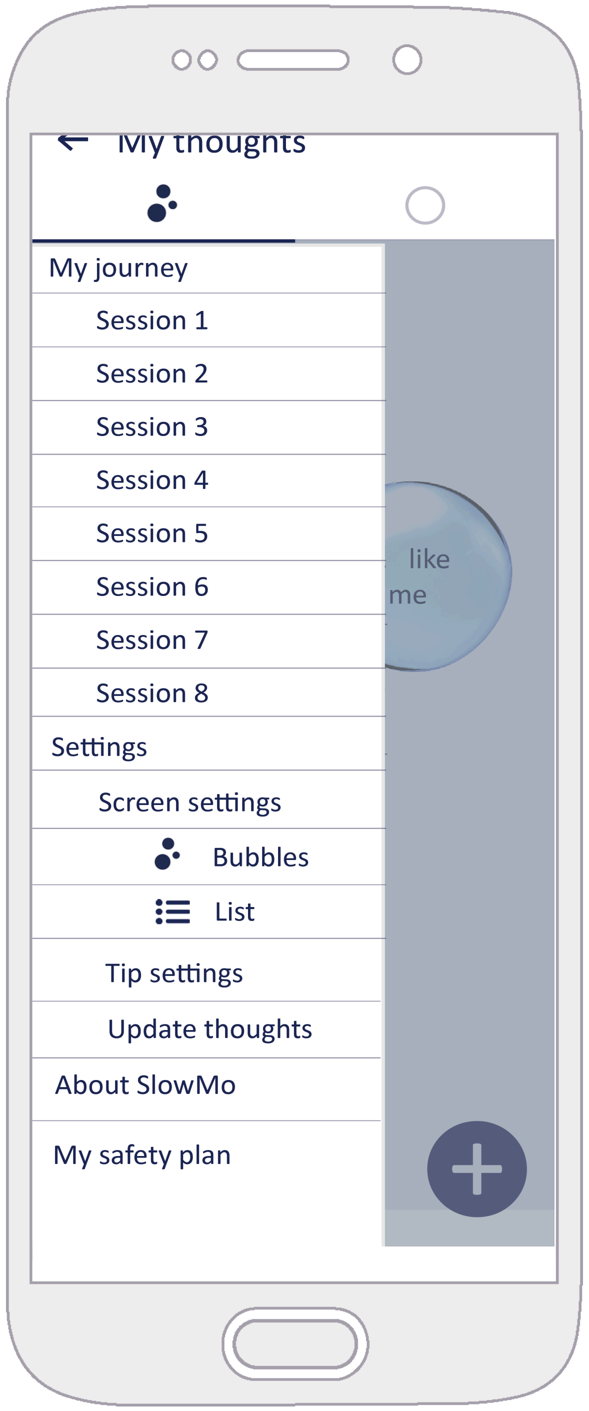

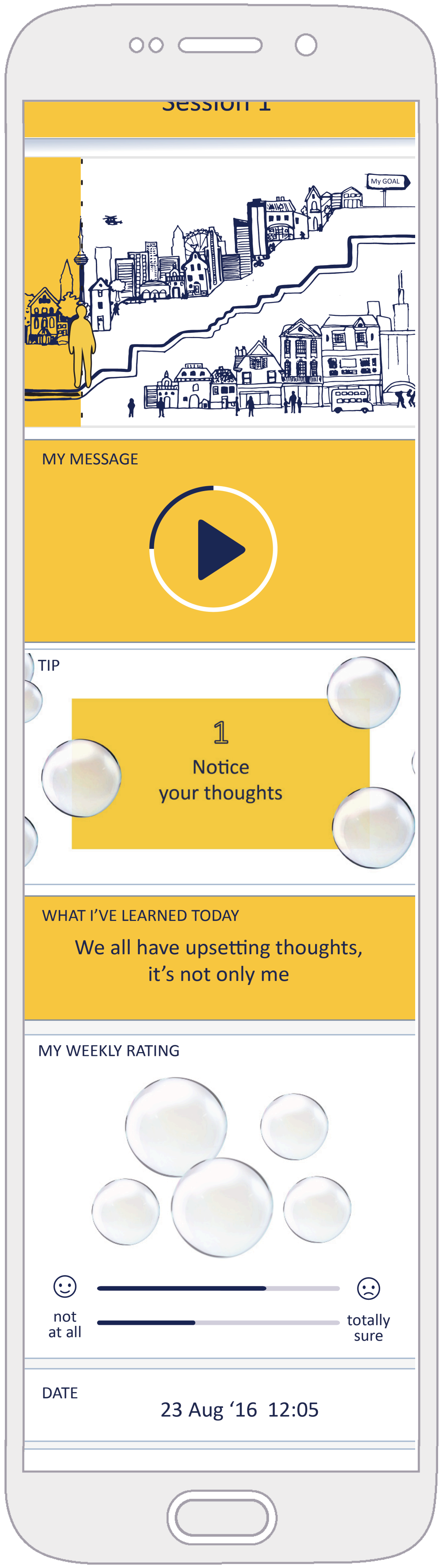

Supplement: Multimedia Appendix 5 [file mental_v5i4e11222_app5.pdf]
